# The association between allergic rhinitis and sleep: A systematic review and meta-analysis of observational studies

**DOI:** 10.1371/journal.pone.0228533

**Published:** 2020-02-13

**Authors:** Jiaomei Liu, Xinge Zhang, Yingying Zhao, Yujiao Wang

**Affiliations:** 1 South Campus of Guang’anmen Hospital, China Academy of Chinese Medical Sciences, Beijing, China; 2 School of Health Sciences, Wuhan University, Wuhan, China; All India Institute of Medical Sciences, Bhopal, INDIA

## Abstract

This systematic review and meta-analysis examines the associations of allergic rhinitis with sleep duration and sleep impairment. Observational studies published before August 2019 were obtained through English language literature searches in the PubMed, Embase, and CINAHL databases. Mean differences and odds ratios with 95% confidence intervals were extracted and used for meta-analysis. Heterogeneity was confirmed by the I^2^-heterogeneity test. Subgroup analysis was conducted to evaluate the influence of study design. The Grading of Recommendations Assessment, Development, and Evaluation approach was used to determine the level of evidence. In total, 2544 records were identified through database searches; 914 duplicate records were excluded, 1452 records were removed after screening of titles and abstracts, 151 records were excluded after full-text screening, and 27 articles were included in the final meta-analyses. A total of 240,706,026 patients (19,444,043 with allergic rhinitis) were considered. No significant difference in sleep duration between the allergic rhinitis and the control groups was found. Patients with allergic rhinitis presented with significantly higher sleep quality scores, sleep disturbances scores, and sleep latency scores; more frequent use of sleep medications; and lower sleep efficiency as measured by the Pittsburgh Sleep Quality Index and polysomnography. Meta-analyses for adjusted odds ratios showed that allergic rhinitis was also associated with higher risks of nocturnal dysfunctions, including insomnia, nocturnal enuresis, restless sleep, sleep-disordered breathing, obstructive sleep apnea, and snoring. Meta-analysis for adjusted odds ratio also showed that allergic rhinitis was associated with daytime dysfunction, including difficulty waking up, daytime sleepiness, morning headache, and the use of sleep medications. The overall quality of evidence ranged from low to very low, indicating that caution is required when interpreting these results. This study demonstrates that there is a significant association of AR with sleep characteristics.

## Introduction

Allergic rhinitis (AR) is a common inflammatory disorder generally caused by an immunoglobulin (Ig) E-mediated response to a variety of environmental allergens, including cockroach frass, animal dander, pollens, dust mites, and molds [1]. It is characterized by two or more symptoms of nasal itching, rhinorrhea, nasal congestion, and/or sneezing [2]. AR has been reported to have an adverse impact on sleep, memory ability, quality of life, academic performance, and work productivity [3–7]. In 2010, AR affected 10%–20% of the worldwide population, and that number continues to grow with modern lifestyle and environmental changes [1]. It is estimated that AR results in 3.4 billion dollars of direct medical costs annually [8].

Sleep is crucial for human mood, memory, endocrine and immune system functions, and cognition [9–14]. Poor sleep quality, sleep disorders, and/or inadequate sleep duration are considered to be triggers for the development of hypertension [15], diabetes [16], obesity [17], cardiovascular disease [18], and increased mortality [19]. AR has been shown to be an important factor in altered sleep patterns in previous studies [7].

There is a growing body of population-based research on the association between AR and sleep patterns. AR has been found to be positively associated with sleep- disordered breathing (SDB) [20], obstructive sleep apnea (OSA) [21], shorter sleep duration [22], poor sleep quality [23], sleep bruxism [24], night sweating [25], nocturnal enuresis [26], and daytime dysfunction [27]. However, these results are also controversial, since several studies found a negative or insignificant relationship between AR and the above sleep-related outcomes [7, 28–30]. Currently, there is a lack of systematic reviews to evaluate the association between AR and sleep pattern through meta-analysis.

Thus, the present study aims to assess the association of AR with sleep pattern by conducting a systematic review and meta-analysis of published observational studies.

## Materials and methods

This systematic review and meta-analysis was carried out in accordance with the Preferred Reporting Items for Systematic Reviews and Meta-Analyses (PRISMA) statement [31].

### Search strategy

Cross-sectional, case-control, and cohort studies examining associations between AR and sleep published before August 2019 were searched in the following databases: PubMed, Embase, and CINAHL. Combinations of sleep-related search terms (“sleep”, “insomnia”, “somnolence”, or “snoring”) and AR-related search terms (“rhinitis”, “rhino conjunctivitis”, “nasal allergy”, or “hay fever”) were employed when screening titles/abstracts/keywords of articles. The full electronic search strategy can be found in the S1 File. Studies that met the inclusion criteria were retrieved for a full-text review, unless the article was unavailable even after every attempt at retrieval. To reduce potential selection bias, each article was independently evaluated by two of the investigators (J.L. and Y.W.), and the final decision to include/exclude was made jointly with a third investigator (Y.Z.) according to the basic inclusion criteria.

### Inclusion criteria

The following inclusion criteria were applied to the literature search process: (1) cross-sectional, case-control, or cohort studies published in refereed English language journals; (2) the study exposure of interest was AR; (3) for studies comparing differences in sleep (sleep quality score, sleep duration, or incidence of adverse sleep events) between AR and a control group, the outcomes were reported as mean + standard deviation (SD), mean + confident interval (CI), mean + standard error (SE), median + interquartile (IQ), median + range, or number of cases with adverse events; (4) for studies evaluating the effect of AR on sleep-related outcomes, estimation of an odds ratio (OR), risk ratio (RR), or hazard ratio (HR) with 95% CI was provided; and (5) study control group was a population without AR.

### Data extraction

Data extraction was performed using a standard data extraction form by one investigator (J.L.) and reviewed by another investigator (Y.W.). Disagreements were resolved through negotiations with a third investigator (Y.Z.) until a consensus was reached. The following information was extracted from included studies: title, last name of the first author, country, number and age of subjects, exposure and outcome, measures of exposure and research endpoints, mean + SD/CI/SE, median + IQ/range, number of cases, and HR/OR/RR with 95% CI.

### Assessment of study quality

The quality of studies included in this review were assessed by the Newcastle-Ottawa Scale (NOS) [32]. A study was defined as high quality if the total stars were six or greater.

### Statistical analysis

We treated HRs as RRs and converted RRs into ORs [33]. Median and CI/SE/IQ/range were converted into mean + SD in accordance with guidelines laid down by Wan *et al* [34]. For studies providing continuous data for comparative analysis between AR and control groups, pooled mean difference (MD) with 95% CI was used as the effect size. Pooled OR with 95% CI was used to evaluate studies reporting OR/RR/HR with 95% CI. Random effects model was used to incorporate included studies [35]. We also performed a fixed effects model to compare studies [36], and the results of this fixed effects model can be found in S1–S5 Figs. Statistical heterogeneity was assessed by the I^2^-heterogeneity test [37], with an I^2^ > 50% indicating high heterogeneity. To evaluate the effects of different study designs on pooled estimates, we performed a subgroup analysis stratified by study design for outcomes with enough included studies. Subgroup analysis stratified by age, patients with OSA, study design, and measurement of outcome was also conducted for sleep duration. For meta-analysis of three or more studies, we omitted one study each time and then calculated the pooled result in order to detect the impact of any one study on the overall results. Since it is suggested that tests for funnel plot asymmetry should be used only when there are at least 10 studies included in the meta-analysis for adequate power, we did not perform the funnel plot or Egger’s test to evaluate publication bias [38]. All statistical analyses were performed through the “meta” package of R software (R Foundation for Statistical Computing, Vienna, Austria).

### Assessment of cumulative evidence

A summary of the overall strength of available evidence was performed using the “Grading of Recommendations Assessment, Development and Evaluation” (GRADE) assessment [39]. Evidence summaries and GRADE assessments were discussed and reviewed by all investigators. A Summary of Findings table was produced by GRADEpro software (McMaster University, Hamilton, Canada) [40].

## Results and discussion

### Literature search and study characteristics

**Fig 1:**
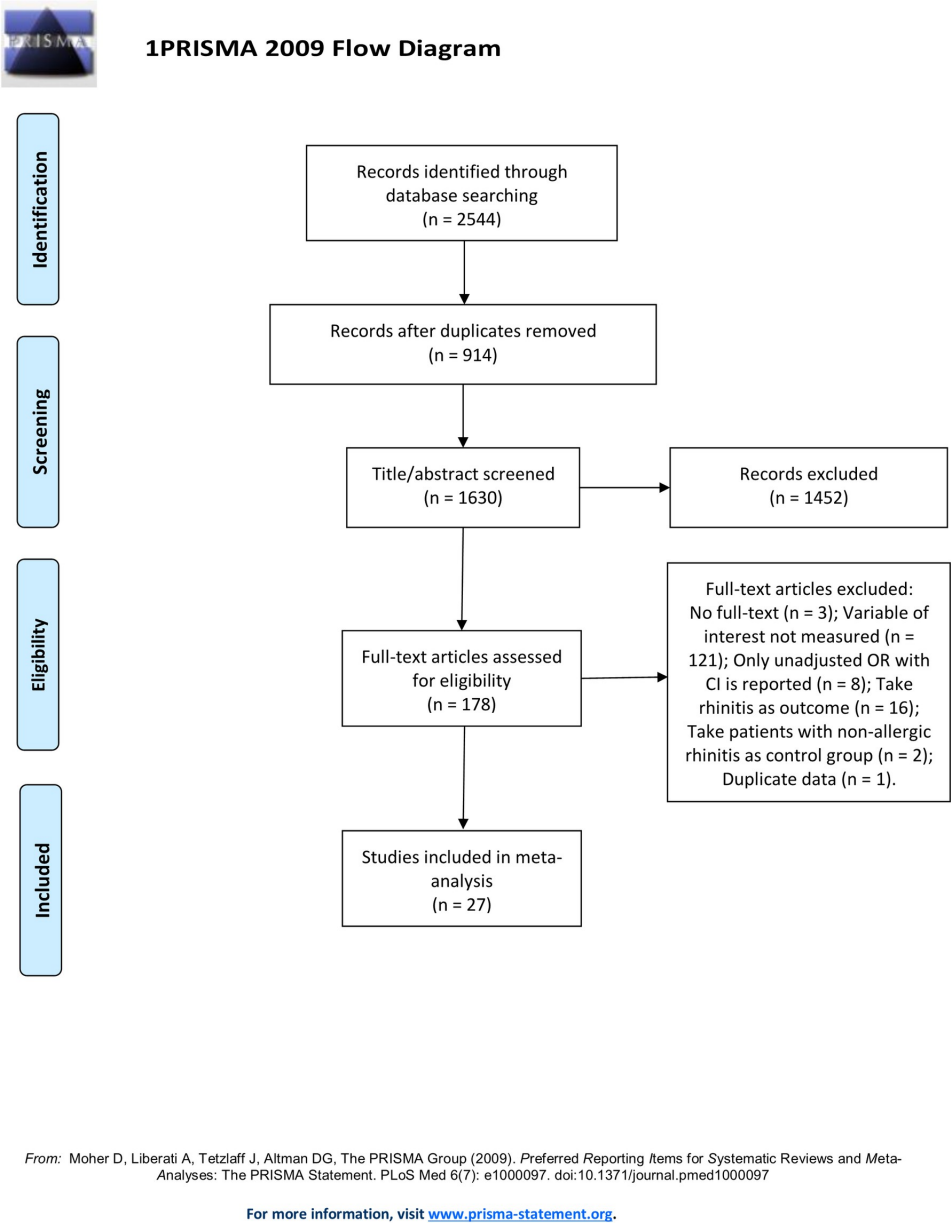
Flow diagram of the literature search and selection processes.

Fig 1 presents the literature search and selection process. 2544 records published before August 2019 were identified. After title, abstract, keyword, and full-text screening, 27 articles were included. The S1 Table lists studies that superficially met the eligibility criteria during full-text screenings; however, failed to meet the criteria on further inspection.

The characteristics of included studies are summarized in Table 1. A total of 240,706,026 patients (19,444,043 AR cases) were included from 13 cross-sectional studies, 10 case-control studies, and 4 cohort studies. Both adult and pediatric patients were included. Medical records, clinical examination findings, skin prick test results, self-reported questionnaires, and face-to-face interviews were reviewed to confirm AR. Sleep–related outcomes were collected and confirmed by medical records, clinical examination findings, self-reported questionnaires, polysomnography (PSG) results, actigraphy results, and face-to-face interviews.

**Table 1:** Characteristics of included studies.

Study	Age	Sample size/AR cases	Study type	Measurement-AR	Measurement-sleep outcome	Outcomes
Roxbury *et al*., 2018	45.6 (44.0–47.11)	5556/1797	Cross-sectional	Self-reported	Self-reported	Sleep duration, SDB, snoring, OSA, insomnia, restless sleep, use of sleeping pills, daytime dysfunction
Loekmanwidjaja *et al*., 2018	4–10	167/112	Case-controlled	Medical records	Self-reported	Sleep duration, daytime sleepiness
Lai *et al*., 2018	<18	655529/327928	Cohort	Medical records	Medical records	OSA, nocturnal enuresis
Filiz *et al*., 2018	8–18	287/143	Cross-sectional	Medical records, clinical examination, and skin prick test	Face-to-face interview	Sleep duration, restless sleep, PSQI outcomes, daytime dysfunction
Zhou *et al*., 2017	47±0.2	240000000/19100000	Cross-sectional	Self-reported	Self-reported	Sleep duration
Zheng *et al*., 2017	18–70	171/65	Cross-sectional	Medical records, clinical examination, and skin prick test	PSG	Sleep duration, OSA, PSG outcomes
Tsai *et al*., 2017	5–18	8516/4191	Case-controlled	Medical records, clinical examination	Medical records	Nocturnal enuresis
Nguyen-Hoang *et al*., 2017	9.5±2.1	85/52	Case-controlled	Clinical examination	Medical records	OSA
Kim *et al*., 2017	68.3±5.6	348/57	Cross-sectional	Self-reported	PSG	SDB, PSG outcomes, PSQI outcomes
Hui *et al*., 2017	49.61±16.27	1028/-	Cross-sectional	Medical records	Medical records	OSA
Di *et al*., 2016	3–14	135/57	Case-controlled	Medical records, clinical examination, and skin prick test	PSG	PSG outcomes
Trikojat *et al*., 2015	18–45	83/41	Case-controlled	Medical records, clinical examination, and skin prick test	Self-reported	Sleep duration, PSQI outcomes, daytime dysfunction
Poachanukoon *et al*., 2015	10.6±2.5	175/65	Case-controlled	Medical records, clinical examination, and skin prick test	Self-reported	Sleep duration, difficult waking up, snoring, morning headache, mouth breathing, night sweating, nocturnal enuresis, OSA daytime sleepiness, restless sleep, SDB, sleep bruxism
Ng *et al*., 2014	15.3±1.7	175/65	Cohort	Self-reported	Self-reported	Snoring
Cai *et al*., 2013	-	1993/123	Cross-sectional	Medical records	Self-reported	OSA
Zhang *et al*., 2012	46.3±5.1	2291/211	Cohort	Self-reported	Self-reported	Restless sleep
So *et al*., 2012	9.2 (7.7–10.7)	6381/2661	Cross-sectional	Self-reported	Self-reported	Night sweating
Park *et al*., 2012	20–68	112/37	Case-controlled	Clinical examination and skin prick test	PSG and self-reported	Sleep duration, AHI, PSG outcomes, ESS
Meng *et al*., 2011	18–60	128/98	Case-controlled	Self-reported	PSG	PSG outcomes
Li *et al*., 2010	5–14	6369/2823	Cross-sectional	Self-reported	Self-reported	Snoring
Rimmer *et al*., 2009	>18	20/10	Case-controlled	Self-reported	Actigraphy	Sleep duration
Hiraki *et al*., 2008	39.4±9.6	852/112	Cross-sectional	Self-reported	Self-reported	Daytime sleepiness, snoring, ESS
Dixon *et al*., 2006	≥15	1969/1133	Cohort	Self-reported	Self-reported	ESS, PSQI outcomes
Sogut *et al*., 2005	8.1±1.9	992/332	Case-controlled	Self-reported	Self-reported	Snoring
Ng *et al*., 2005	6–12	3047/1242	Cross-sectional	Self-reported	Self-reported	Daytime sleepiness, OSA
Chng *et al*., 2004	4–7	9362/674	Cross-sectional	Self-reported	Self-reported	Snoring
Anuntaseree *et al*., 2001	7.25±0.58	255/14	Cross-sectional	Self-reported	Self-reported	Snoring

AR: allergic rhinitis; ESS: Epworth Sleepiness Scale; HS: habitual snoring; NAR: non-allergic rhinitis; OSA: obstructive sleep apnea; PSG: polysomnography; PSQI: Pittsburgh Sleep Quality Index; REM: rapid eye movement; SDB: sleep-disordered breathing.

### Comparative analyses of sleep outcomes between AR patients and controls

**Fig 2:**
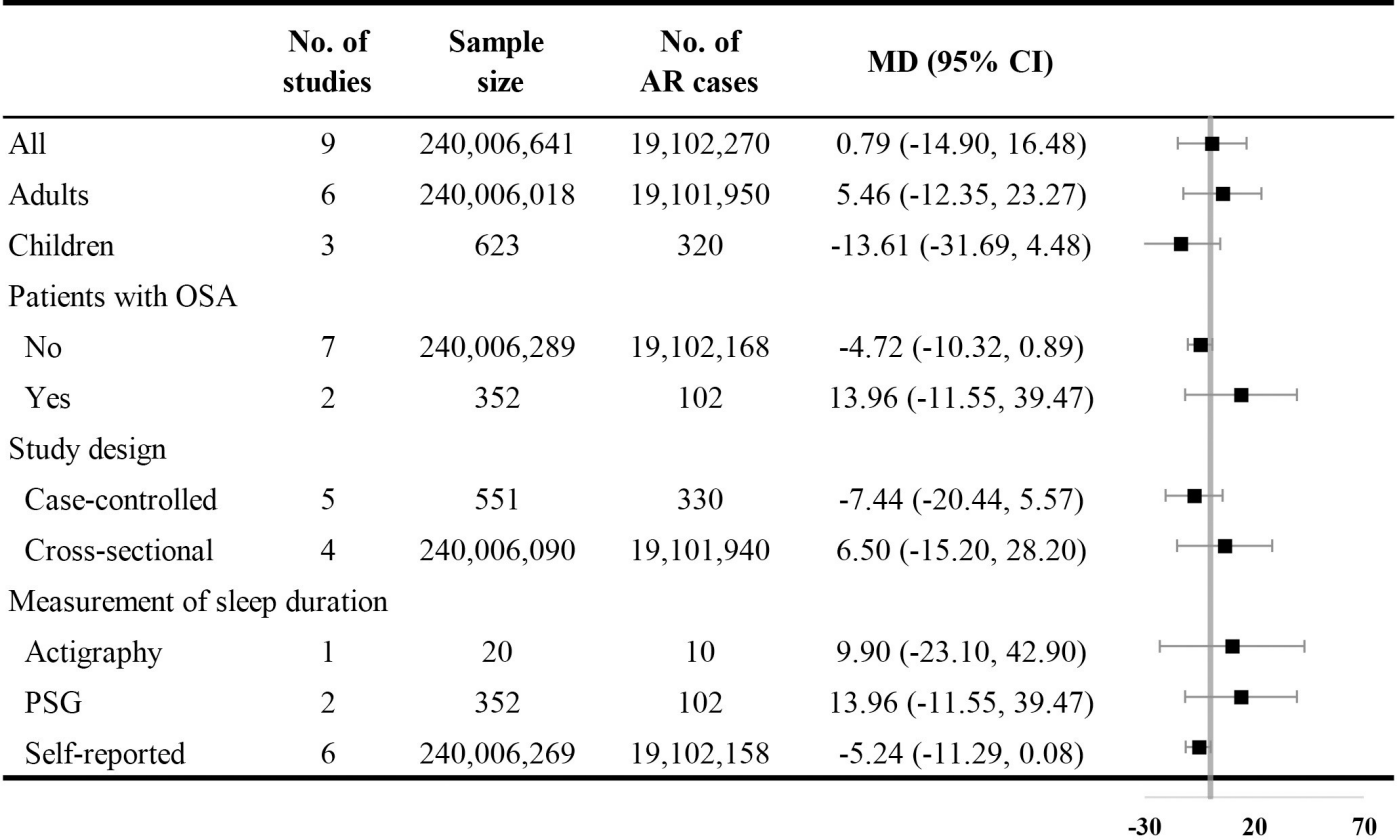
Comparative analysis of sleep duration. AR: allergic rhinitis; CI: confidence interval; MD: mean difference; OSA: obstructive sleep apnea; PSG: polysomnography.

Nine studies reported sleep durations of AR and control groups. Pooled results showed no significant difference in sleep duration between the groups [MD with 95% CI = 0.79 (-14.90, 16.48)] with high heterogeneity (I^2^ = 93.00%). Subgroup analysis stratified by age, presence of OSA, study design, and measurement of sleep duration were conducted, but no significant differences were observed (Fig 2).

**Fig 3:**
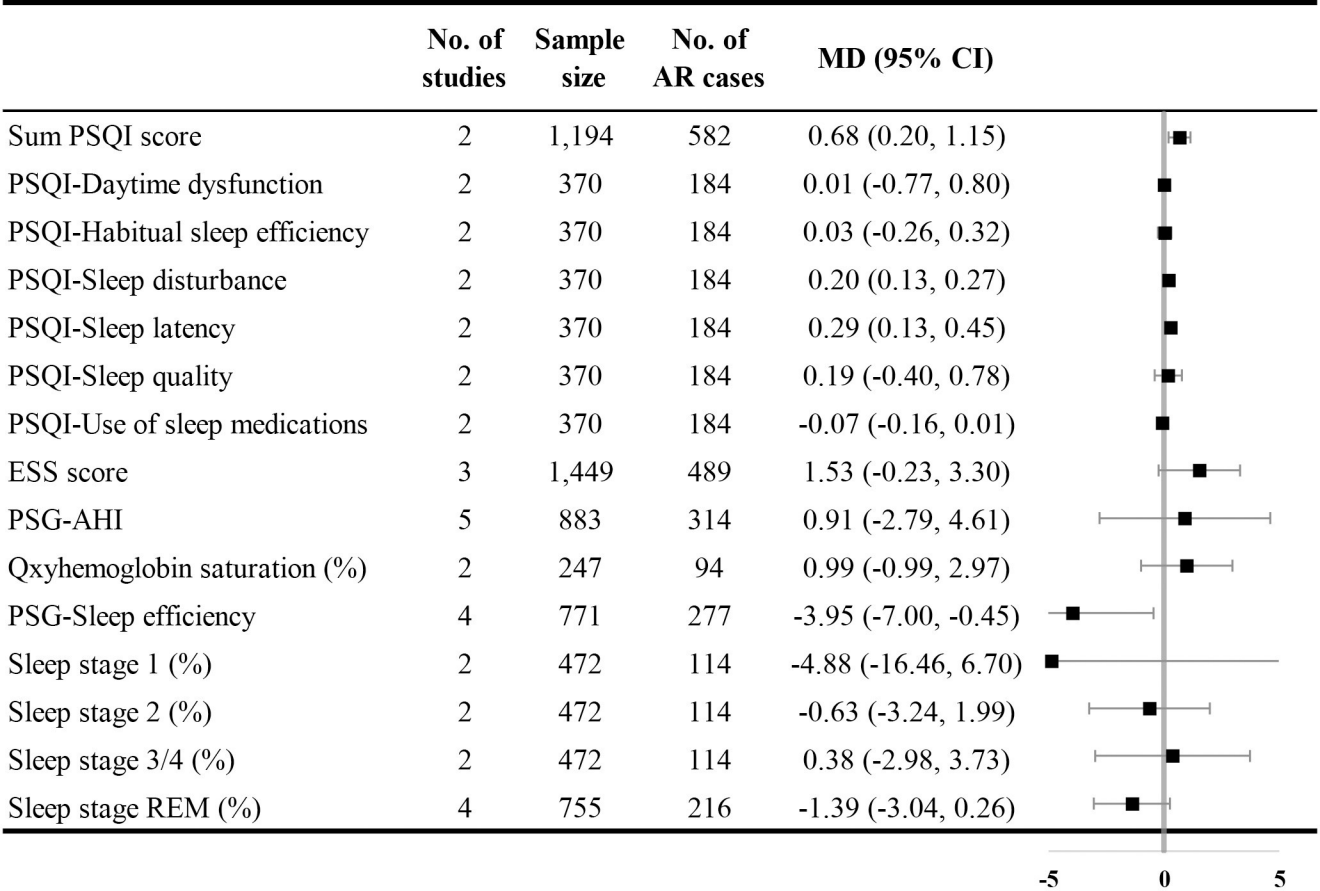
Comparative analysis of PSQI scale, ESS scale, and PSG outcomes. AHI: apnea-hypopnea index; ESS: Epworth Sleepiness Scale; PSQI: Pittsburgh Sleep Quality Index; REM: rapid eye movement.

Four studies reported comparisons of Pittsburgh Sleep Quality Index (PSQI) scores in patients with AR. Compared with the control group, the AR group presented with higher sum PSQI scores [MD with 95% CI = 0.68 (0.20, 1.15)], higher sleep disturbances scores [MD with 95% CI = 0.20 (0.13, 0.27)], and higher sleep latency scores (MD with 95% CI = 0.29 (0.13, 0.45)). The I^2^ was 20.90% for the summed PSQI score and 0.00% for the sleep disturbance and sleep latency scores. Three studies provided comparisons of the Epworth Sleepiness Scale (ESS) in patients with AR. The AR group presented with a higher, though not significant, sum ESS score [MD with 95% CI = 1.53 (-0.23, 3.30)]. Five articles reporting PSG outcomes showed lower scores of sleep efficiency [MD with 95% CI = -3.95 (-7.00, -0.45), I^2^ = 89.00%] in the AR group (Fig 3).

### Adjusted associations between AR and sleep outcomes

**Fig 4:**
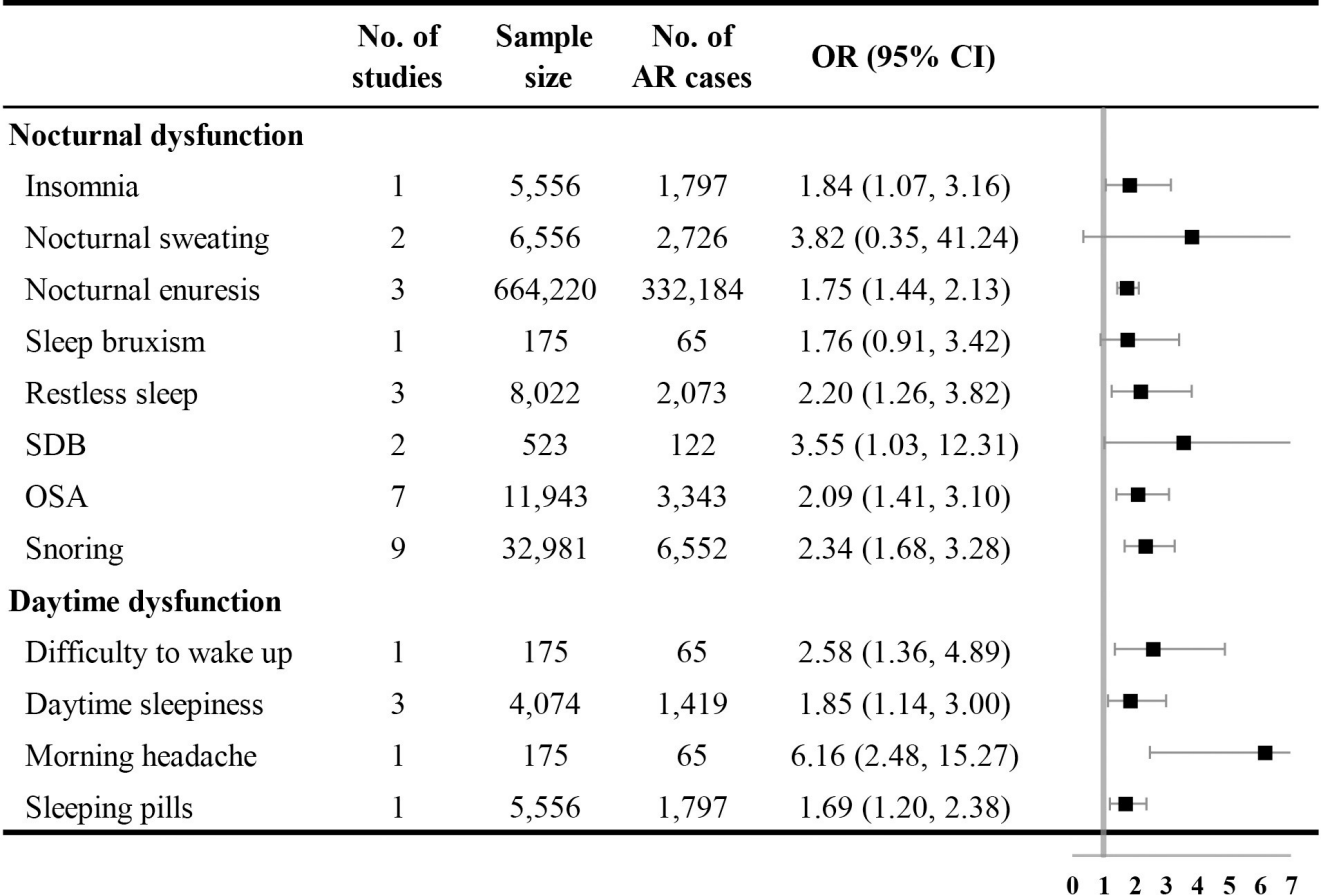
Forest plot for adjusted associations between AR and sleep outcomes. AR: allergic rhinitis; CI: confidence interval; OR: odds ratio; OSA: obstructive sleep apnea; SDB: sleep-disordered breathing.

Fig 4 shows the adjusted relationship between AR and sleep outcomes. Patients with AR were more likely to suffer from nocturnal sleep-related dysfunctions, including insomnia [OR with 95% CI = 1.84 (1.07, 3.16)], nocturnal enuresis [OR with 95% CI = 1.75 (1.44, 2.13), I^2^ = 28.50%], restless sleep [OR with 95% CI = 2.20 (1.26, 3.82), I^2^ = 51.20%], SDB [OR with 95% CI = 3.55 (1.03, 12.31), I^2^ = 85.80%], OSA [OR with 95% CI = 2.09 (1.41, 3.10), I^2^ = 53.60%], and snoring [OR with 95% CI = 2.34 (1.68, 3.28), I^2^ = 83.40%]. AR was also associated with a higher risk of daytime sleep-related dysfunctions, including difficulty waking up [OR with 95% CI = 2.58 (1.36, 4.89)], daytime sleepiness [OR with 95% CI = 1.85 (1.14, 3.00), I^2^ = 53.20%], morning headache [OR with 95% CI = 6.16 (2.48, 15.27)], and the use of sleeping pills [OR with 95% CI = 1.69 (1.20, 2.38)].

### Evaluation of the effect of study design

We conducted subgroup analysis stratified by study design for sleep duration, apnea-hypopnea index (AHI), sleep efficiency measured by PSG, the percent of sleep stage REM measured by PSG, OSA, and snoring. Significant differences in results from different study designs were observed for AHI, sleep efficiency measured by PSG, and the percent of sleep stage REM measured by PSG, indicating the potential influence of study design on pooled results. Detailed information is shown in the supplemental material (S6–S11 Figs).

### Assessment of study quality and sensitivity analysis

Most of the included studies were of high quality according to the NOS criteria. Detailed information is shown in S2–S4 Tables.

By omitting one study each time using a random effects model, sensitivity analysis was conducted for meta-analyses of sleep duration, ESS score, AHI assessed by PSG, sleep efficiency assessed by PSG, the percentage of sleep stage REM assessed by PSG, nocturnal enuresis, restless sleep, OSA, snoring, and daytime sleepiness. Pooled results of sleep duration and AHI assessed by PSG were significantly sensitive to the study of Zheng *et al* [30], because the direction of the synthesized MD changed after it was omitted. The results of the ESS score, sleep efficiency evaluated by PSG, nocturnal enuresis, restless sleep, and daytime sleepiness also showed instability. Detailed information is presented in S12–S21 Figs.

### Assessment of cumulative evidence

The overall quality of evidence using GRADE’s Summary of Findings table was judged to be low or very low. Risk of bias, inconsistencies (methodological, clinical, and statistical), indirectness, and imprecision were found due to lack of adjusting confounding factors, incorporation of different age groups and study designs, self-reported AR, and sleep outcomes. The Summary of Findings table can be found in S5 and S6 Tables.

In the present review, no significant differences in sleep duration between the AR and control groups were observed. AR patients presented with increased sleep quality scores, sleep disturbance scores, and sleep latency scores on the PSQI scale and a decreased sleep efficiency score using PSG. AR was also found to be associated with a higher risk of nocturnal sleep-related dysfunctions, including insomnia, nocturnal enuresis, restless sleep, SDB, OSA, and snoring. Additionally, AR was found to be associated with a higher risk of daytime sleep-related dysfunctions, including difficulty waking up, daytime sleepiness, morning headache, and the use of sleeping pills.

The main underlying mechanisms for the association between AR and altered sleep patterns can be summarized as follows: (1) inflammatory cytokines related to AR produce fatigue directly; (2) AR symptoms and underlying pathophysiologic changes affect sleep indirectly; and (3) the effect of autonomic system dysfunction in patients with AR.

Inflammatory mediators, including histamine, are released in AR and have a direct influence on the central nervous system, contributing to sleep disturbances and daytime sleepiness [41, 42]. Histamine can also affect the regulation of the sleep-wake cycle, which may result in arousal disorder [41, 43]. AR also induces decreased levels of the interleukins (ILs)-1β, IL-4, IL-6 and IL-10, which may increase REM sleep (important in the restorative function of sleep), decrease sleep onset latency, improve circadian rhythm, and regulate slow wave sleep [44]. These changes contribute to why patients with AR often have difficulties with overnight sleep and daytime tiredness.

Symptoms of AR include nasal congestion, rhinorrhea, sneezing, and pruritus. Among these symptoms, nasal obstruction is the most troublesome one for patients [45–47]. Nasal congestion is considered to be a major factor interfering with sleep quality and inducing daytime somnolence. Previous studies suggest that nasal obstruction contributes to higher risks of OSA [48–50] and SDB [51, 52]. The role of nasal congestion as a risk factor for snoring has also been confirmed by a population-based cohort study [53]. A negative effect of nasal congestion was also found on quality of life and daytime productivity [54, 55]. Data on sleep-related end points from clinical trials of nasal decongestants are associated with improved sleep, reduced daytime fatigue, and improved quality of life [56]. Other symptoms including cough and sputum production also contribute to poor sleep quality and sleep disorders [57, 58].

Imbalance of the autonomic system in AR is also thought to be involved to the association between AR and sleep impairment [59]. As one of the most powerful autonomic nerve reflexes, the trigeminocardiac reflex (TCR) is believed to directly influence the development of SDB, OSA, REM sleep apnea, and nasal congestion [60–62]. The nasotrigeminal reflex, a peripheral nervous system equivalent of the TCR, is also thought to have a potential influence on sleep impairment [60]. The extent of autonomic dysfunction in AR and sleep disturbances is still not well-established and needs further investigation.

To the best of our knowledge, this is the first systematic review and meta-analysis evaluating the association between AR and sleep based on observational investigations. A 2018 systematic review focused on the association between AR and OSA and applied a meta-analysis of population-based studies; however, this article concentrated mainly on the prevalence of AR in subjects with or without OSA/SDB [63]. Another related review examined AR and sleep impairment, but focused mainly on the mechanism of their association and the consequences of disordered sleep [64–71].

There are some limitations to the current study. First, most of the included studies are cross-sectional or case-controlled, which do not allow for inferences of causal relationships. Second, some results from this study’s meta-analyses display sensitivity to a single study included in the analysis, potentially due to the limited number of included studies. In terms of the sensitivity analysis of sleep duration and AHI assessed by PSG, the inverse MD is caused by the study of Zheng *et al*., which was conducted in patients with OSA. We included a limited number of studies focusing on OSA patients and failed to detect a difference between the normal population and patients with OSA. More investigations conducted in patients with OSA are needed. Third, some outcomes of sleep impairment are confirmed by self-reported questionnaires, which are vulnerable to recall bias and potentially affected by social norms. Further longitudinal research with objective measurements are warranted.

## Conclusions

There is a significant association of AR with sleep characteristics; however, due to the very low GRADE level of evidence, caution is required when interpreting our results.

## Supporting information

S1 Fig Forest plot for sleep duration. CI: confidence interval; MD: mean difference; SD: standard deviation.
(TIF)

S2 Fig Forest plot for PSQI scale. CI: confidence interval; MD: mean difference; PSQI: Pittsburgh Sleep Quality Index; SD: standard deviation.
(TIF)

S3 Fig Forest plot for ESS score. CI: confidence interval; ESS: Epworth Sleepiness Scale; MD: mean difference; SD: standard deviation.
(TIF)

S4 Fig Forest plot for PSG outcomes. AHI: apnea-hypopnea index; CI: confidence interval; ESS: Epworth Sleepiness Scale; MD: mean difference; PSG: polysomnography; REM: rapid eye movement; SD: standard deviation.
(TIF)

S5 Fig Forest plot for adjusted association between AR and sleep outcomes. AR: allergic rhinitis; CI: confidence interval; OR: odds ratio; OSA: obstructive sleep apnea; SDB: sleep-disordered breathing.
(TIF)

S6 Fig Subgroup analysis by study design for sleep duration. CI: confidence interval; MD: mean difference; SD: standard deviation.
(TIF)

S7 Fig Subgroup analysis by study design for AHI. AHI: apnea-hypopnea index; CI: confidence interval; MD: mean difference; SD: standard deviation.
(TIF)

S8 Fig Subgroup analysis by study design for sleep efficiency measured by PSG. AHI: apnea-hypopnea index; CI: confidence interval; MD: mean difference; PSG: polysomnography; SD: standard deviation.
(TIF)

S9 Fig Subgroup analysis by study design for sleep stage REM (%). CI: confidence interval; MD: mean difference; REM: rapid eye movement; SD: standard deviation.
(TIF)

S10 Fig Subgroup analysis by study design for OSA. CI: confidence interval; OR: odds ratio; OSA: obstructive sleep apnea.
(TIF)

S11 Fig Subgroup analysis by study design for snoring. CI: confidence interval; OR: odds ratio.
(TIF)

S12 Fig Sensitivity analysis for sleep duration. CI: confidence interval; MD: mean difference.
(TIFF)

S13 Fig Sensitivity analysis for ESS score. CI: confidence interval; ESS: Epworth Sleepiness Scale; MD: mean difference.
(TIFF)

S14 Fig Sensitivity analysis for AHI assessed by PSG. AHI: apnea-hypopnea index; CI: confidence interval; MD: mean difference; PSG: polysomnography.
(TIFF)

S15 Fig Sensitivity analysis for sleep efficiency assessed by PSG. CI: confidence interval; MD: mean difference; PSG: polysomnography.
(TIFF)

S16 Fig Sensitivity analysis for sleep stage REM (%) assessed by PSG. CI: confidence interval; MD: mean difference; PSG: polysomnography; REM: rapid eye movement.
(TIFF)

S17 Fig Sensitivity analysis for nocturnal enuresis. CI: confidence interval; OR: odds ratio.
(TIFF)

S18 Fig Sensitivity analysis for restless sleep. CI: confidence interval; OR: odds ratio.
(TIFF)

S19 Fig Sensitivity analysis for OSA. CI: confidence interval; OR: odds ratio; OSA: obstructive sleep apnea.
(TIFF)

S20 Fig Sensitivity analysis for snoring. CI: confidence interval; OR: odds ratio.
(TIFF)

S21 Fig Sensitivity analysis for daytime sleepiness. CI: confidence interval; OR: odds ratio.
(TIFF)

S1 Table Excluded studies from analysis. (DOCX)

S2 Table Assessment of the quality of cross-sectional studies using NOS. (DOCX)

S3 Table Assessment of the quality of case-controlled studies using NOS. (DOCX)

S4 Table Assessment of the quality of cohort studies using NOS. (DOCX)

S5 Table Summary of Findings for continuous outcomes. AHI: apnea-hypopnea index; CI: confidence interval; ESS: Epworth Sleepiness Scale; MD: mean difference; REM: rapid eye movement; PSQI: Pittsburgh Sleep Quality Index; PSG: polysomnography.
(DOCX)

S6 Table Summary of Findings for dichotomous outcomes. CI: confidence interval; MD: mean difference; OR: odds ratio; OSA: obstructive sleep apnea; SD: standard deviation; SDB: sleep-disordered breathing.
(DOCX)

S7 Table PRISMA checklist. (DOCX)

S1 File Electronic search strategy. (TXT)
