# Supplementary figures and images for: The association between allergic rhinitis and sleep: A systematic review and meta-analysis of observational studies
Source: PLoS One. 2020 Feb 13;15(2):e0228533. doi: 10.1371/journal.pone.0228533 (PMC7018032; doi:10.1371/journal.pone.0228533)

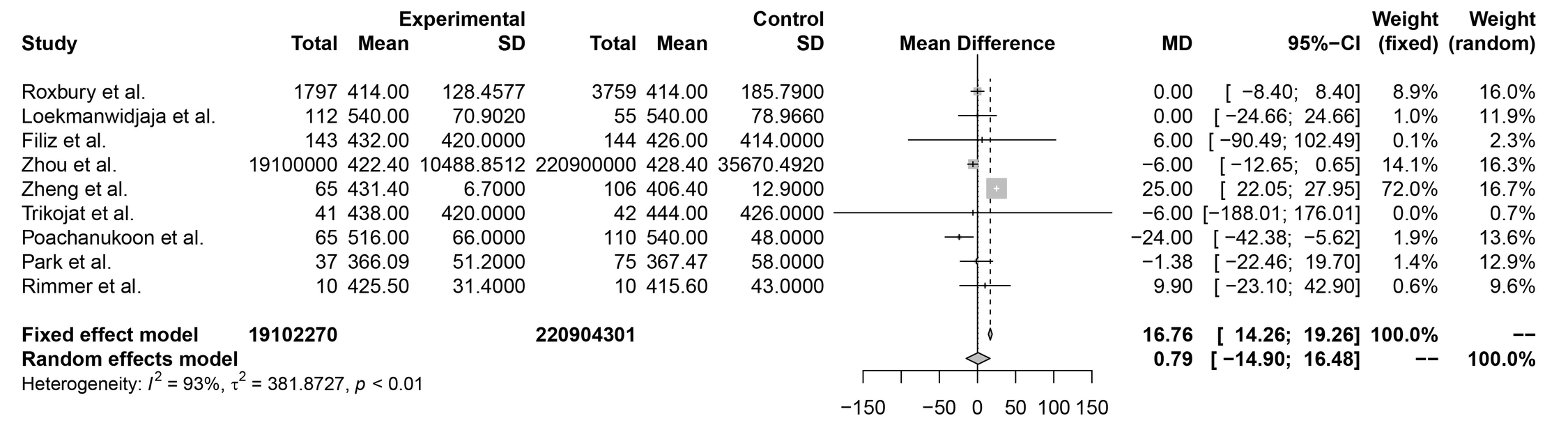

Supplement: S1 Fig — CI: confidence interval; MD: mean difference; SD: standard deviation. (TIF) [file pone.0228533.s001.tif]

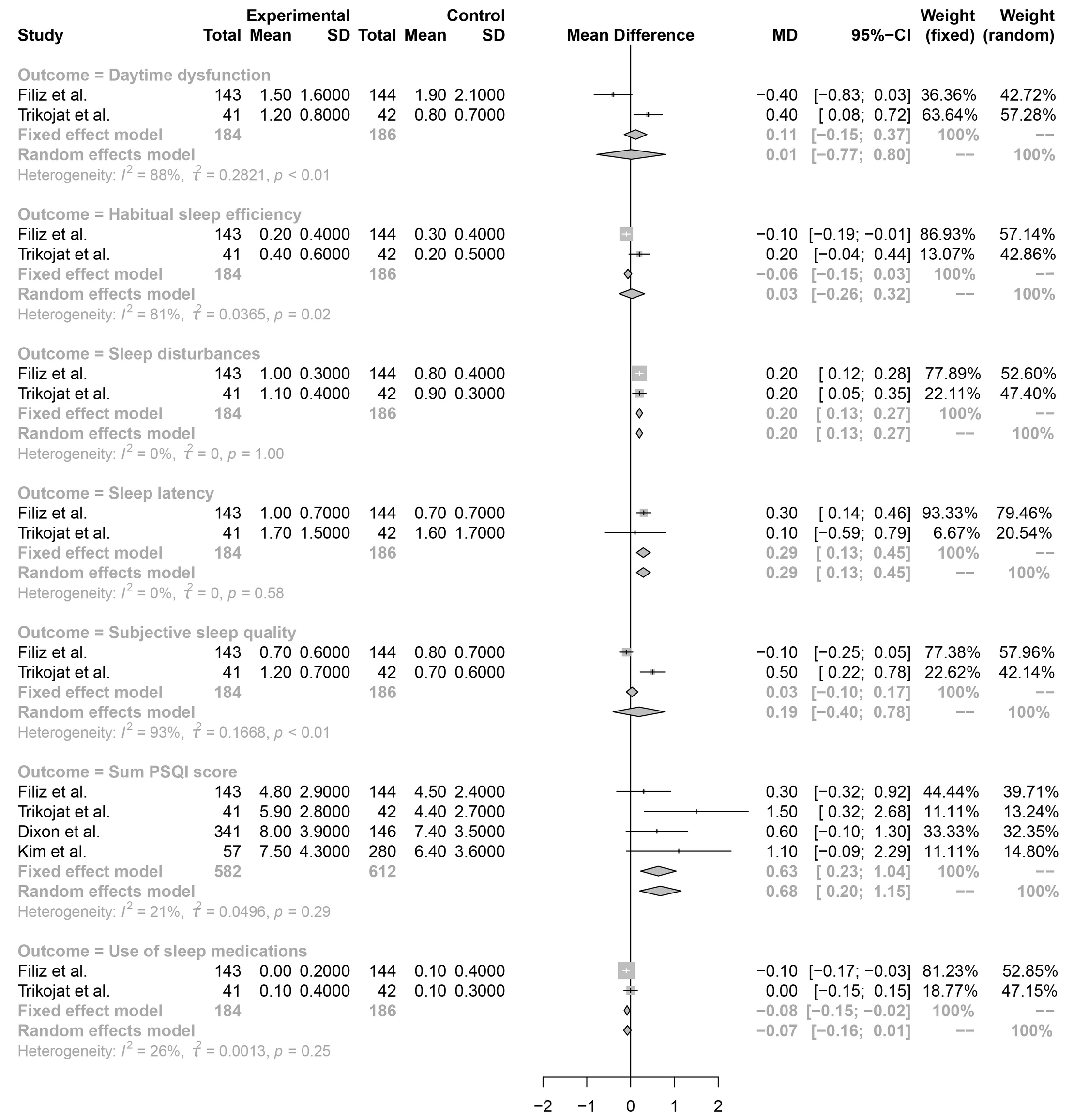

Supplement: S2 Fig — CI: confidence interval; MD: mean difference; PSQI: Pittsburgh Sleep Quality Index; SD: standard deviation. (TIF) [file pone.0228533.s002.tif]

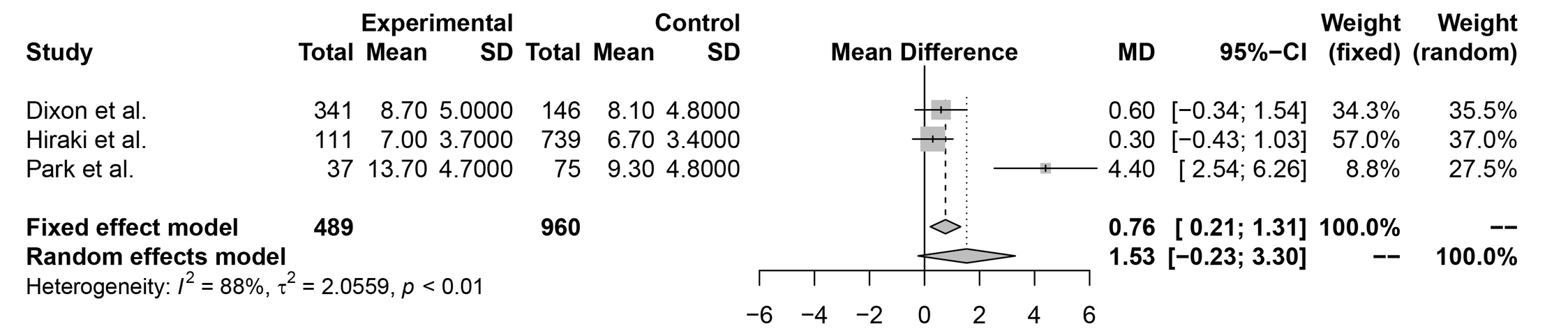

Supplement: S3 Fig — CI: confidence interval; ESS: Epworth Sleepiness Scale; MD: mean difference; SD: standard deviation. (TIF) [file pone.0228533.s003.tif]

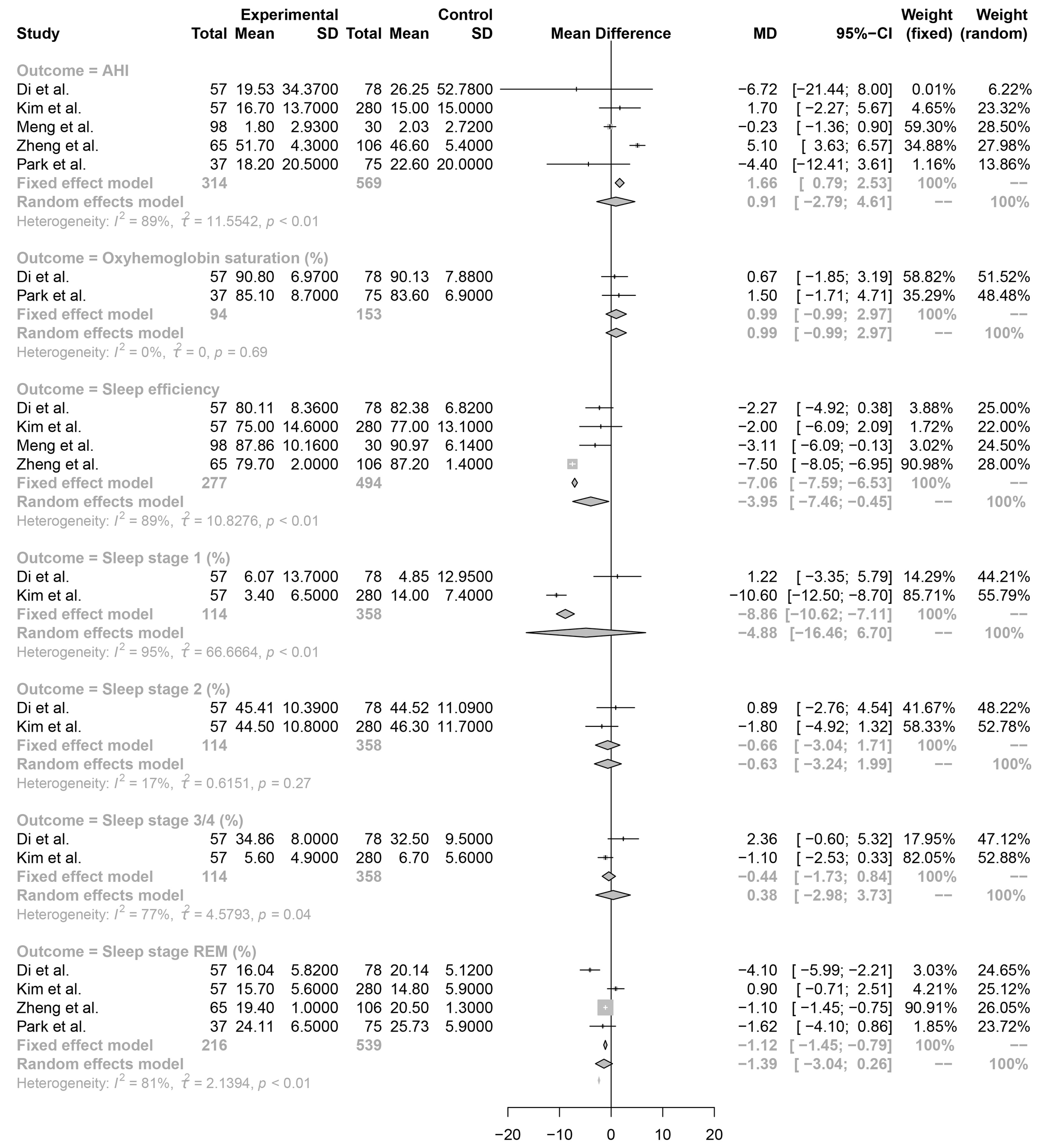

Supplement: S4 Fig — AHI: apnea-hypopnea index; CI: confidence interval; ESS: Epworth Sleepiness Scale; MD: mean difference; PSG: polysomnography; REM: rapid eye movement; SD: standard deviation. (TIF) [file pone.0228533.s004.tif]

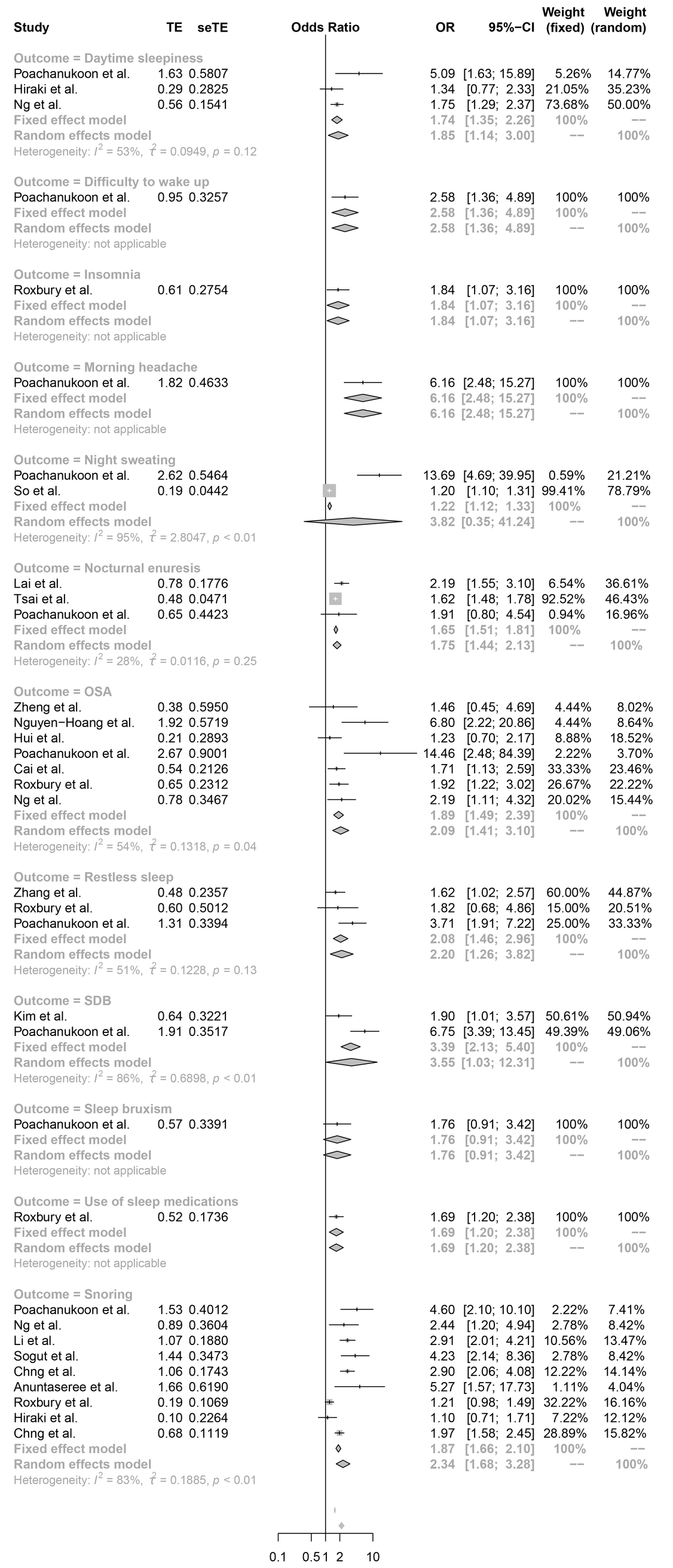

Supplement: S5 Fig — AR: allergic rhinitis; CI: confidence interval; OR: odds ratio; OSA: obstructive sleep apnea; SDB: sleep-disordered breathing. (TIF) [file pone.0228533.s005.tif]

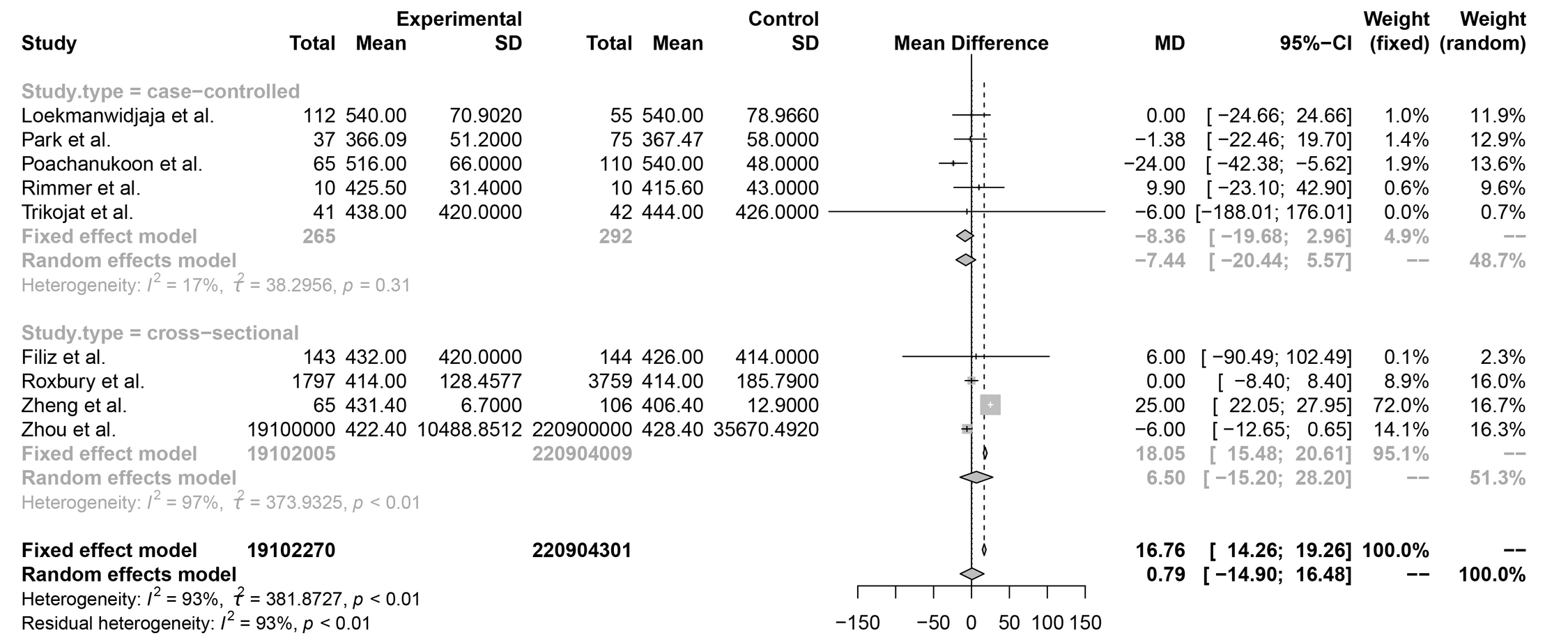

Supplement: S6 Fig — CI: confidence interval; MD: mean difference; SD: standard deviation. (TIF) [file pone.0228533.s006.tif]

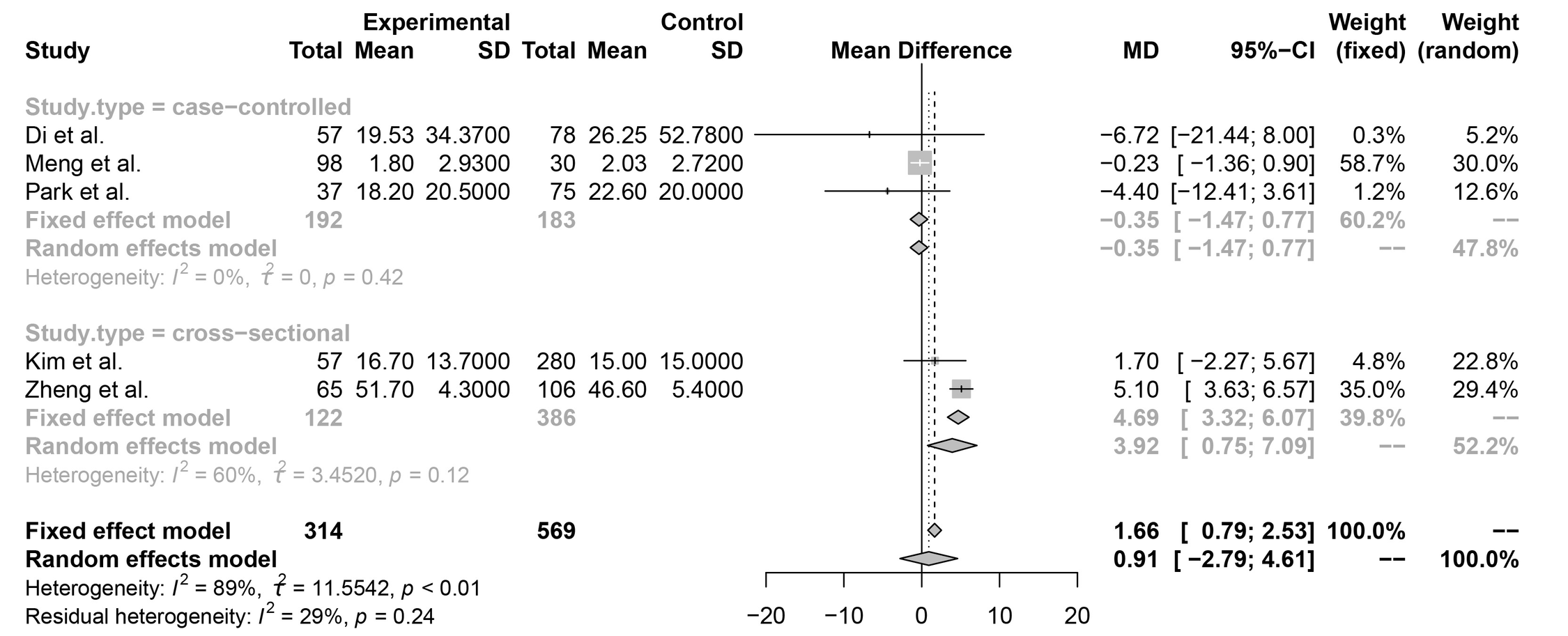

Supplement: S7 Fig — AHI: apnea-hypopnea index; CI: confidence interval; MD: mean difference; SD: standard deviation. (TIF) [file pone.0228533.s007.tif]

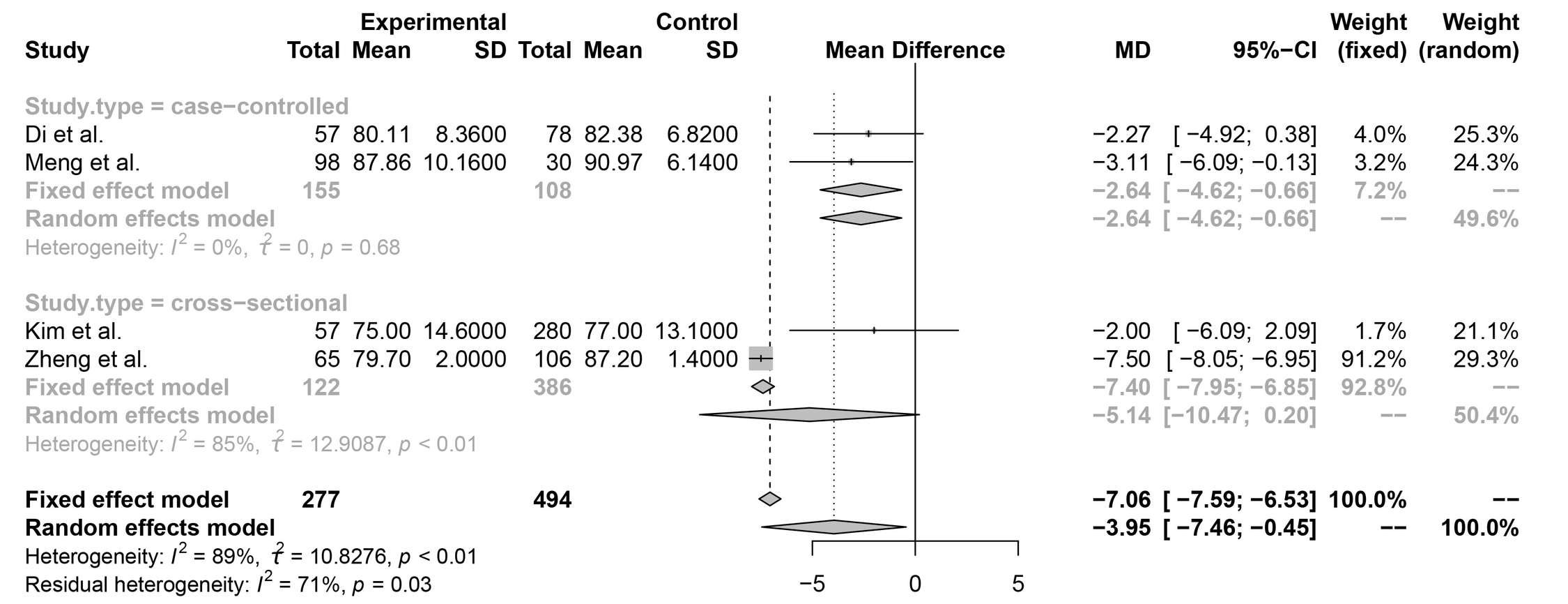

Supplement: S8 Fig — AHI: apnea-hypopnea index; CI: confidence interval; MD: mean difference; PSG: polysomnography; SD: standard deviation. (TIF) [file pone.0228533.s008.tif]

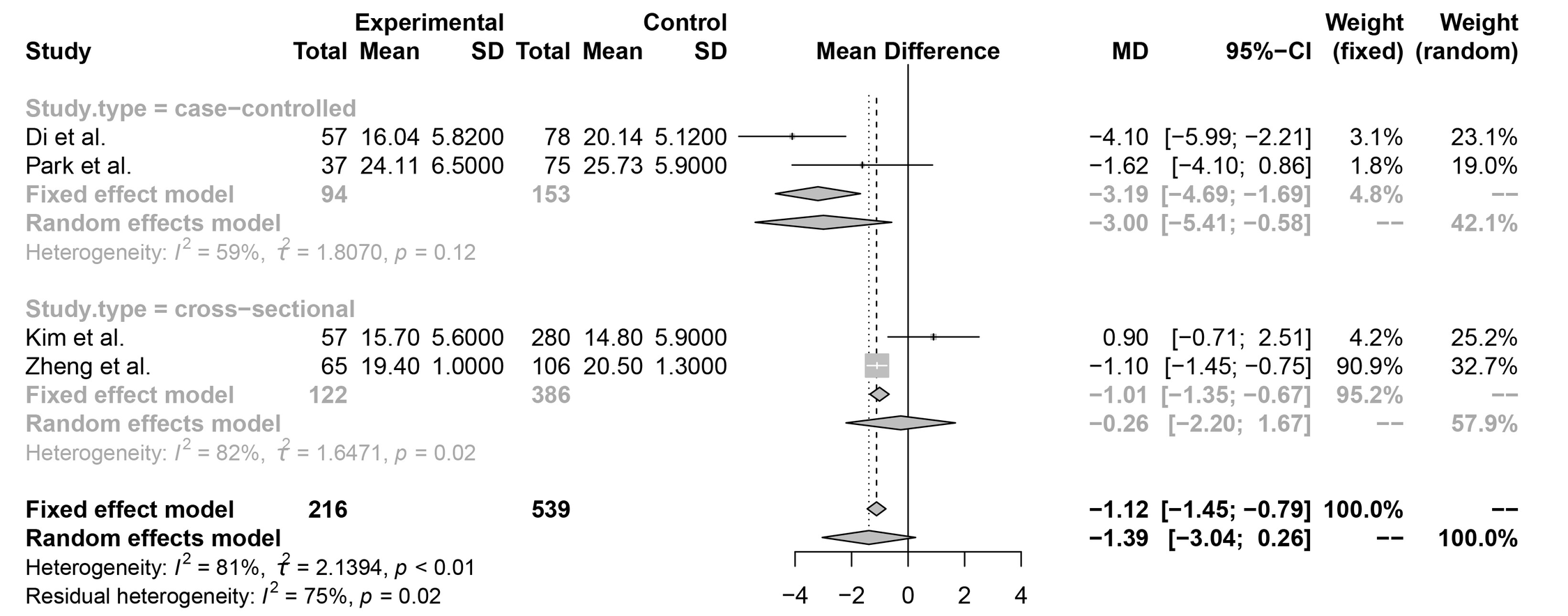

Supplement: S9 Fig — CI: confidence interval; MD: mean difference; REM: rapid eye movement; SD: standard deviation. (TIF) [file pone.0228533.s009.tif]

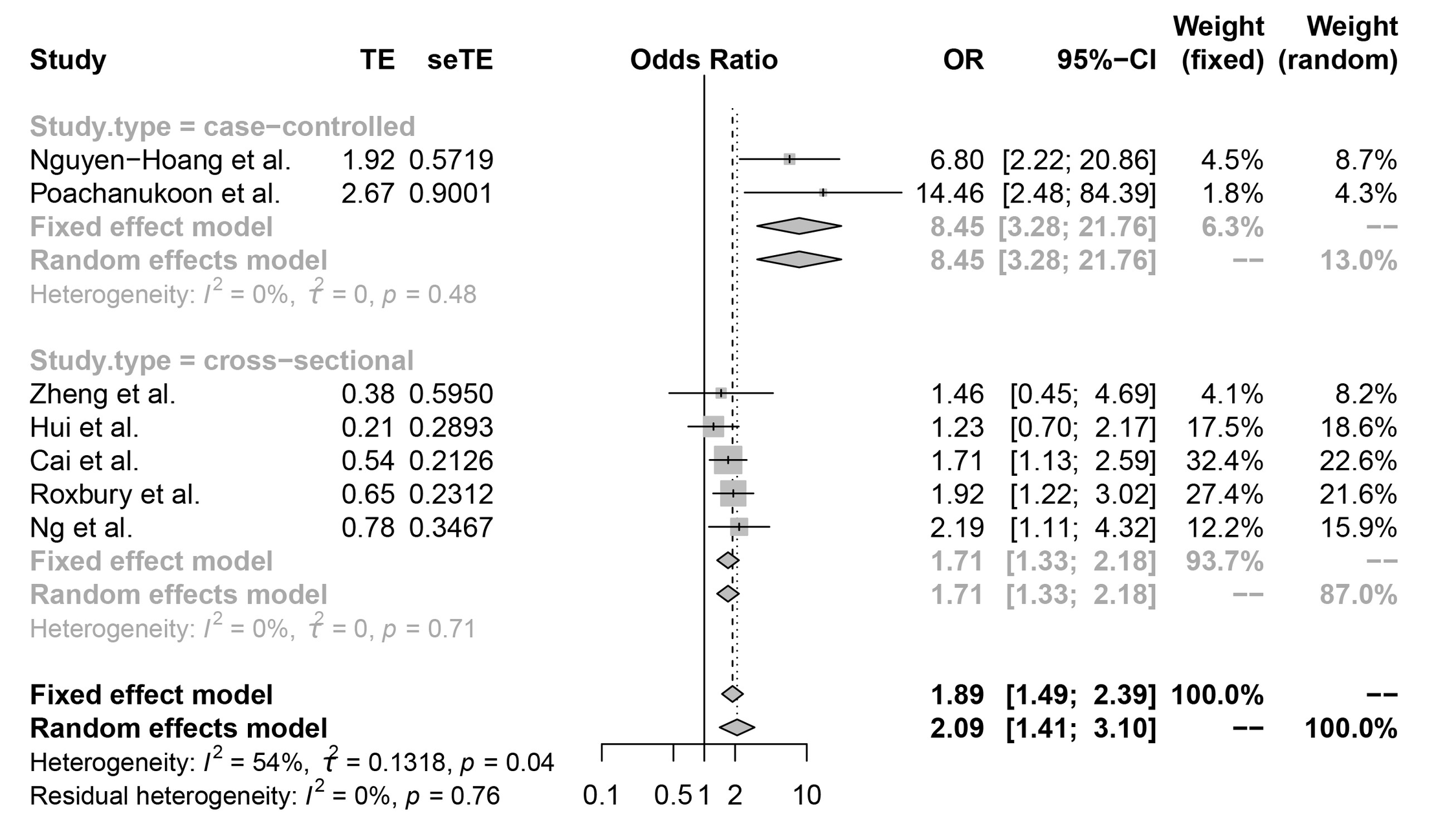

Supplement: S10 Fig — CI: confidence interval; OR: odds ratio; OSA: obstructive sleep apnea. (TIF) [file pone.0228533.s010.tif]

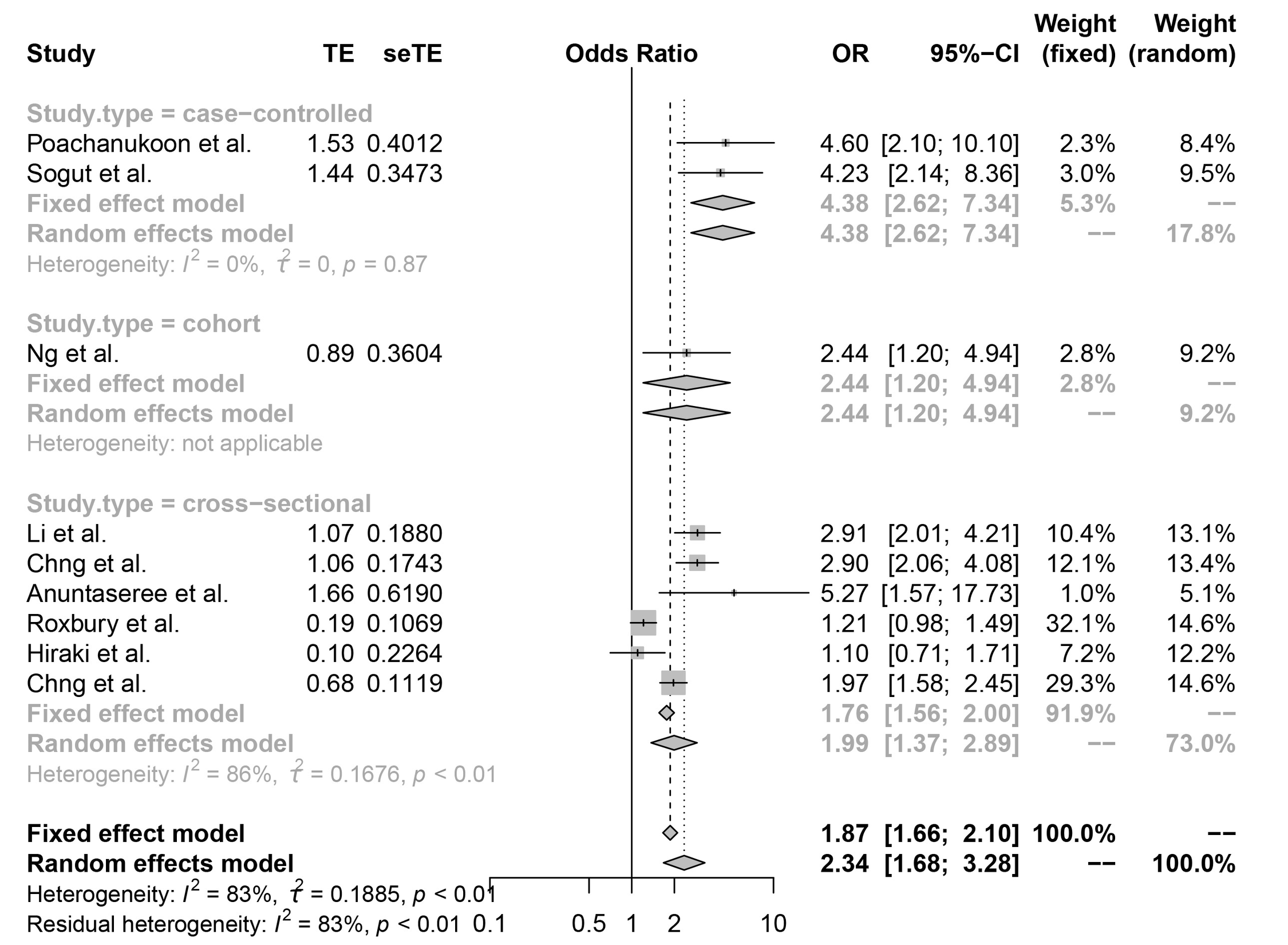

Supplement: S11 Fig — CI: confidence interval; OR: odds ratio. (TIF) [file pone.0228533.s011.tif]

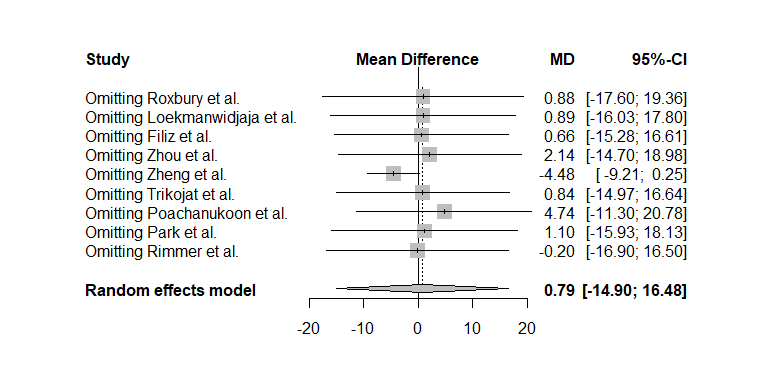

Supplement: S12 Fig — CI: confidence interval; MD: mean difference. (TIFF) [file pone.0228533.s012.tiff]

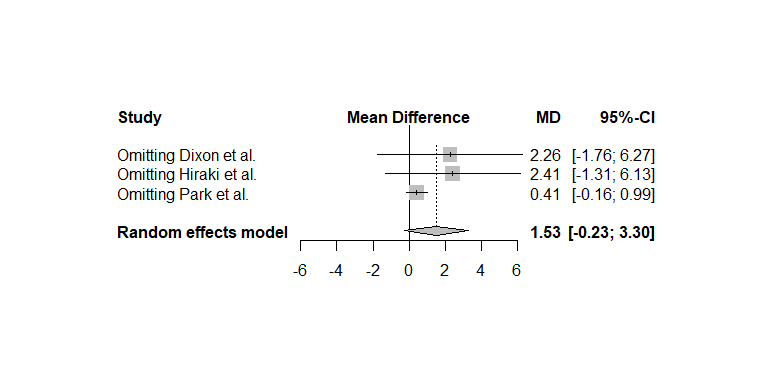

Supplement: S13 Fig — CI: confidence interval; ESS: Epworth Sleepiness Scale; MD: mean difference. (TIFF) [file pone.0228533.s013.tiff]

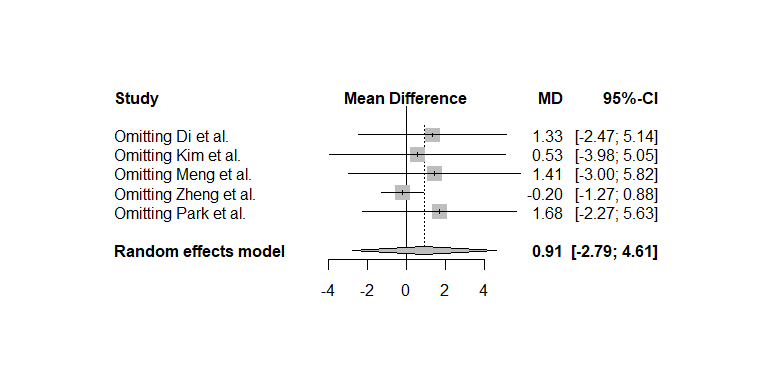

Supplement: S14 Fig — AHI: apnea-hypopnea index; CI: confidence interval; MD: mean difference; PSG: polysomnography. (TIFF) [file pone.0228533.s014.tiff]

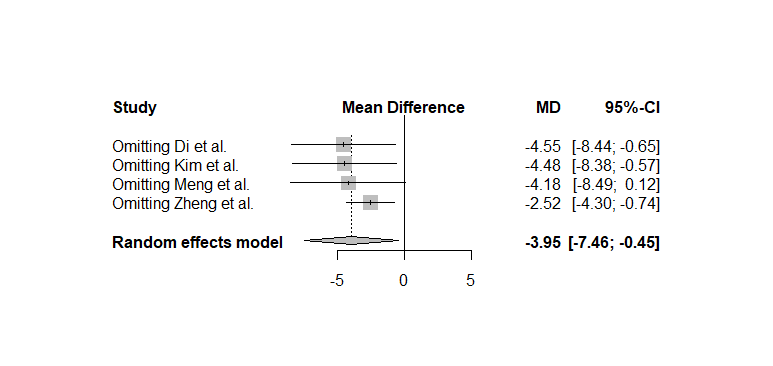

Supplement: S15 Fig — CI: confidence interval; MD: mean difference; PSG: polysomnography. (TIFF) [file pone.0228533.s015.tiff]

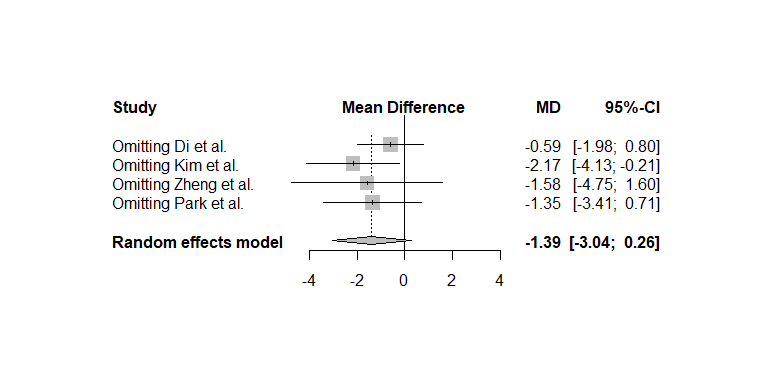

Supplement: S16 Fig — CI: confidence interval; MD: mean difference; PSG: polysomnography; REM: rapid eye movement. (TIFF) [file pone.0228533.s016.tiff]

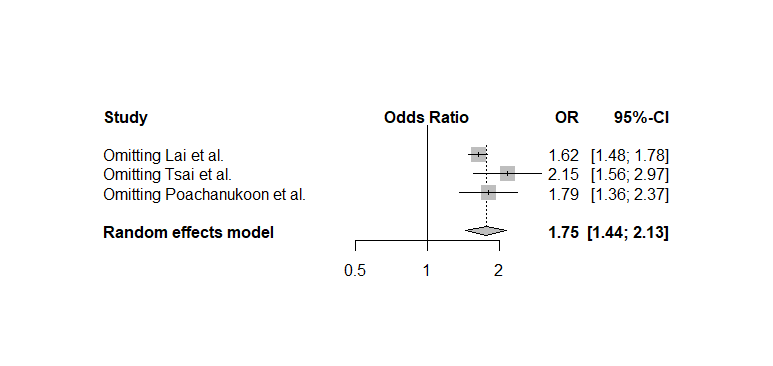

Supplement: S17 Fig — CI: confidence interval; OR: odds ratio. (TIFF) [file pone.0228533.s017.tiff]

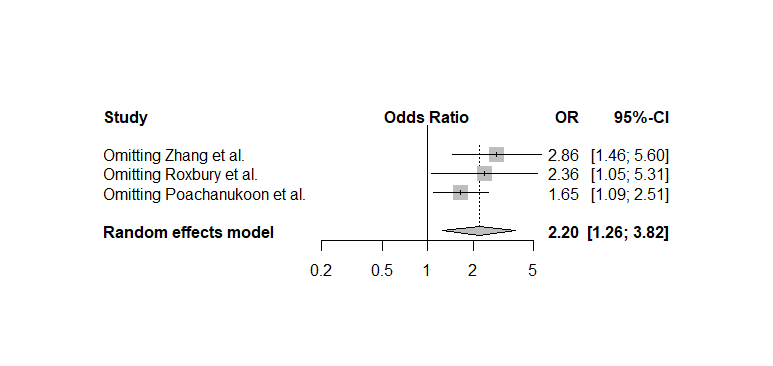

Supplement: S18 Fig — CI: confidence interval; OR: odds ratio. (TIFF) [file pone.0228533.s018.tiff]

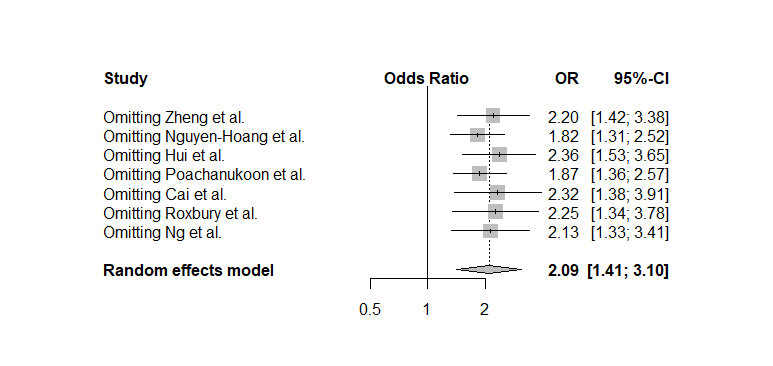

Supplement: S19 Fig — CI: confidence interval; OR: odds ratio; OSA: obstructive sleep apnea. (TIFF) [file pone.0228533.s019.tiff]

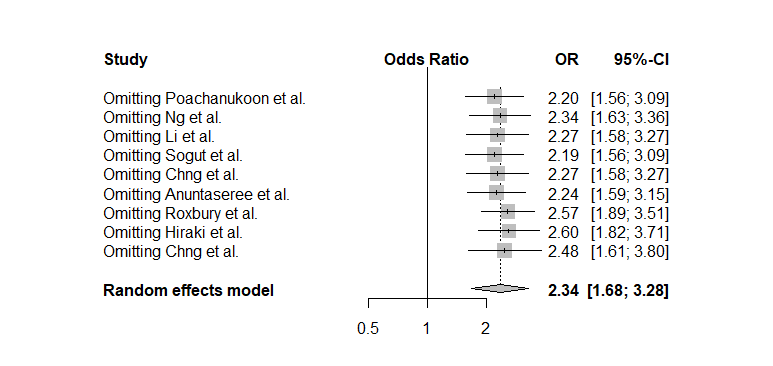

Supplement: S20 Fig — CI: confidence interval; OR: odds ratio. (TIFF) [file pone.0228533.s020.tiff]

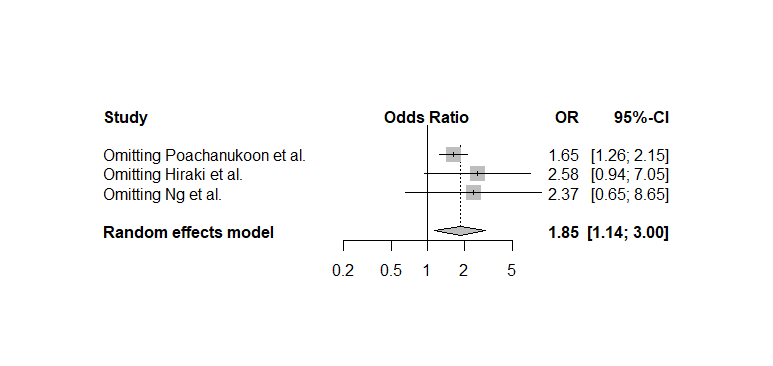

Supplement: S21 Fig — CI: confidence interval; OR: odds ratio. (TIFF) [file pone.0228533.s021.tiff]
