# Supplementary material for: The association between allergic rhinitis and sleep: A systematic review and meta-analysis of observational studies
Source: PLoS One. 2020 Feb 13;15(2):e0228533. doi: 10.1371/journal.pone.0228533 (PMC7018032; doi:10.1371/journal.pone.0228533)
Supplement: S1 Table — (DOCX) [file pone.0228533.s022.docx]

**S1 Table. Excluded studies from analysis.**

| **Excluded studies** | **Rationale for exclusion** |
| --- | --- |
| 1. Braido, F., I. Baiardini, D. Lacedonia, F. M. Facchini, F. Fanfulla, G. Molinengo & G. W. Canonica (2014) Sleep apnea risk in subjects with asthma with or without comorbid rhinitis. Respir Care, 59, 1851-6. | Take rhinitis as outcome. |
| 1. Desager, K. N., V. Nelen, J. J. Weyler & W. A. De Backer (2005) Sleep disturbance and daytime symptoms in wheezing school-aged children. J Sleep Res, 14, 77-82. | Take chronic rhinitis as outcome. |
| 1. Drumond, C. L., D. S. Souza, J. M. Serra-Negra, L. S. Marques, M. L. Ramos-Jorge & J. Ramos-Jorge (2017) Respiratory disorders and the prevalence of sleep bruxism among schoolchildren aged 8 to 11 years. Sleep and Breathing, 21, 203-208. | Take rhinitis as outcome. |
| 1. Gadi, G., S. Wali, E. Koshak, M. Albar, A. Fida, M. Abdelaziz, K. Alnoury & N. Alama (2017) The prevalence of allergic rhinitis and atopic markers in obstructive sleep apnea. J Epidemiol Glob Health, 7, 37-44. | Take patients with non-allergic rhinitis as control group, |
| 1. Hellgren, J., E. Omenaas, T. Gislason, R. Jogi, K. A. Franklin, E. Lindberg, C. Janson & K. Toren (2007) Perennial non-infectious rhinitis--an independent risk factor for sleep disturbances in Asthma. Respir Med, 101, 1015-20. | Take rhinitis as outcome. |
| 1. Huseni, S., M. J. Gutierrez, C. E. Rodriguez-Martinez, C. L. Nino, G. F. Perez, K. Pancham & G. Nino (2014) The link between rhinitis and rapid-eye-movement sleep breathing disturbances in children with obstructive sleep apnea. Am J Rhinol Allergy, 28, e56-61. | Take rhinitis as outcome. |
| 1. Janson, C., W. De Backer, T. Gislason, P. Plaschke, E. Bjornsson, J. Hetta, H. Kristbjarnarson, P. Vermeire & G. Boman (1996) Increased prevalence of sleep disturbances and daytime sleepiness in subjects with bronchial asthma: a population study of young adults in three European countries. Eur Respir J, 9, 2132-8. | Take rhinitis as outcome. |
| 1. Jernelov, S., M. Lekander, C. Almqvist, J. Axelsson & H. Larsson (2013) Development of atopic disease and disturbed sleep in childhood and adolescence--a longitudinal population-based study. Clin Exp Allergy, 43, 552-9. | Take rhinitis as outcome. |
| 1. Kalpaklioǧlu, A. F., A. B. Kavut & M. Ekici (2009) Allergic and nonallergic rhinitis: The threat for obstructive sleep apnea. Annals of Allergy, Asthma and Immunology, 103, 20-25. | Take patients with non-allergic rhinitis as control group, |
| 1. Krakow, B., M. Foley-Shea, V. A. Ulibarri, N. D. McIver & R. Honsinger (2016) Prevalence of potential nonallergic rhinitis at a community-based sleep medical center. Sleep Breath, 20, 987-93. | Only unadjusted OR with CI is reported. |
| 1. Larsson, L. G., A. Lindberg, K. A. Franklin & B. Lundback (2001) Symptoms related to obstructive sleep apnoea are common in subjects with asthma, chronic bronchitis and rhinitis in a general population. Respir Med, 95, 423-9. | Take rhinitis as outcome. |
| 1. Lee, K. S., H. Y. Yum, Y. H. Sheen, Y. M. Park, Y. J. Lee, B. S. Choi, H. M. Jee, S. H. Choi, H. H. Kim, Y. Park, H. B. Kim & Y. H. Rha (2017) Comorbidities and phenotypes of rhinitis in Korean children and adolescents: A cross-sectional, multicenter study. Allergy, Asthma and Immunology Research, 9, 70-78. | Take rhinitis as outcome. |
| 1. Léger, D., I. Annesi-Maesano, F. Carat, M. Rugina, I. Chanal, C. Pribil, A. El Hasnaoui & J. Bousquet (2006) Allergic rhinitis and its consequences on quality of sleep: An unexplored area. Archives of Internal Medicine, 166, 1744-1748. | Only unadjusted OR with CI is reported. |
| 1. Li, S., X. Jin, C. Yan, S. Wu, F. Jiang & X. Shen (2010) Habitual snoring in school-aged children: environmental and biological predictors. Respir Res, 11, 144. | Only unadjusted OR with CI is reported. |
| 1. Martikainen, K., M. Partinen, J. Hasan, P. Laippala, H. Urponen & I. Vuori (2003) The impact of somatic health problems on insomnia in middle age. Sleep Medicine, 4, 201-206. | Take rhinitis as outcome. |
| 1. Meltzer, E. O., M. S. Blaiss, M. J. Derebery, T. A. Mahr, B. R. Gordon, K. K. Sheth, A. L. Simmons, M. A. Wingertzahn & J. M. Boyle (2009) Burden of allergic rhinitis: results from the Pediatric Allergies in America survey. J Allergy Clin Immunol, 124, S43-70. | Only unadjusted OR with CI is reported. |
| 1. Ng, T. P. & W. C. Tan (2005) Prevalence and determinants of excessive daytime sleepiness in an Asian multi-ethnic population. Sleep Medicine, 6, 523-529. | Take rhinitis as outcome. |
| 1. Schatz, M., R. S. Zeiger, W. Chen, S. J. Yang, M. A. Corrao & V. P. Quinn (2008) The burden of rhinitis in a managed care organization. Ann Allergy Asthma Immunol, 101, 240-7. | Take rhinitis as outcome. |
| 1. Sogut, A., O. Yilmaz, G. Dinc & H. Yuksel (2009) Prevalence of habitual snoring and symptoms of sleep-disordered breathing in adolescents. Int J Pediatr Otorhinolaryngol, 73, 1769-73. | Only unadjusted OR with CI is reported. |
| 1. Tafur, A., I. Chérrez-Ojeda, C. Patiño, D. Gozal, C. Rand, M. Ronnie, G. Thomas, S. Jaime & C. Jacquelin (2009) Rhinitis symptoms and habitual snoring in Ecuadorian children. Sleep Medicine, 10, 1035-1039. | Take rhinitis as outcome. |
| 1. Tamanyan, K., L. M. Walter, M. J. Davey, G. M. Nixon, R. S. C. Horne & S. N. Biggs (2016) Risk factors for obstructive sleep apnoea in Australian children. Journal of Paediatrics and Child Health, 52, 512-517. | Only unadjusted OR with CI is reported. |
| 1. Tien, K. J., C. W. Chou, S. Y. Lee, N. C. Yeh, C. Y. Yang, F. C. Yen, J. J. Wang & S. F. Weng (2014) Obstructive sleep apnea and the risk of atopic dermatitis: a population-based case control study. PLoS One, 9, e89656. | Only unadjusted OR with CI is reported. |
| 1. Trikojat, K., H. Luksch, A. Rösen-Wolff, F. Plessow, J. Schmitt & A. Buske-Kirschbaum (2017) “Allergic mood” – Depressive and anxiety symptoms in patients with seasonal allergic rhinitis (SAR) and their association to inflammatory, endocrine, and allergic markers. Brain, Behavior, and Immunity, 65, 202-209. | Data was included in Trikojat, K., et al. (2015) |
| 1. Urrutia-Pereira, M., D. Solé, H. J. Chong Neto, V. Acosta, A. M. Cepeda, M. Álvarez-Castelló, C. F. Almendarez, J. Lozano-Saenz, J. C. Sisul-Alvariza, N. A. Rosario, A. J. Castillo, M. Valentin-Rostan, H. Badellino, R. L. Castro-Almarales, M. González-León, C. Sanchez-Silot, M. M. Avalos, C. Fernandez, F. Berroa, M. M. De la Cruz & R. O. S. Sarni (2017) Sleep disorders in Latin-American children with asthma and/or allergic rhinitis and normal controls. Allergologia et Immunopathologia, 45, 145-151. | Take rhinitis as outcome. |
| 1. Verhulst, S. L., K. Vekemans, E. Ho, L. Aerts, S. Jacobs, L. A. De Backer, H. Dhanapala, P. Perera, W. A. De Backer & K. N. Desager (2007) Is wheezing associated with decreased sleep quality in Sri Lankan children? A questionnaire study. Pediatr Pulmonol, 42, 579-83. | Take rhinitis as outcome. |
| 1. Vichyanond, P., C. Suratannon, P. Lertbunnaphong, O. Jirapongsananuruk & N. Visitsunthorn (2010) Clinical characteristics of children with non-allergic rhinitis vs with allergic rhinitis. Asian Pacific Journal of Allergy and Immunology, 28, 270-274. | Take rhinitis as outcome. |
| 1. Wang, X., X. Gao, Q. Yang, X. Wang, S. Li, F. Jiang, J. Zhang & F. Ouyang (2017) Sleep disorders and allergic diseases in Chinese toddlers. Sleep Medicine, 37, 174-179. | Only unadjusted OR with CI is reported. |
