# Supplementary material for: The association between allergic rhinitis and sleep: A systematic review and meta-analysis of observational studies
Source: PLoS One. 2020 Feb 13;15(2):e0228533. doi: 10.1371/journal.pone.0228533 (PMC7018032; doi:10.1371/journal.pone.0228533)
Supplement: S2 Table — (DOCX) [file pone.0228533.s023.docx]

**S2 Table. Assessment of cross-sectional studies quality.**

|  | **Representativeness of the sample** | **Sample size** | **Non-respondents** | **Ascertainment of the exposure (risk factor)** | **Comparability** | **Assessment of the outcome** | **Statistical test** | **Total score** |
| --- | --- | --- | --- | --- | --- | --- | --- | --- |
| Roxbury *et al*., 2018 | ● | ● | ● | ●○ | ●○ | ●○ | ● | 7 |
| Filiz *et al*., 2018 | ● | ○ | ● | ●● | ●○ | ●● | ● | 8 |
| Zhou *et al*., 2017 | ● | ● | ● | ●○ | ●○ | ●○ | ● | 7 |
| Zheng *et al*., 2017 | ● | ○ | ● | ●● | ●○ | ●● | ● | 8 |
| Kim *et al*., 2017 | ● | ○ | ● | ●● | ●○ | ●○ | ● | 7 |
| Hui *et al*., 2017 | ● | ● | ● | ●● | ●○ | ●● | ● | 9 |
| Cai *et al*., 2013 | ● | ● | ● | ●○ | ●○ | ●● | ● | 8 |
| So *et al*., 2012 | ● | ● | ● | ●○ | ●○ | ●○ | ● | 7 |
| Li *et al*., 2010 | ● | ● | ● | ●○ | ●○ | ●○ | ● | 7 |
| Hiraki *et al*., 2008 | ● | ○ | ● | ●○ | ●○ | ●○ | ● | 6 |
| Ng *et al*., 2005 | ● | ● | ● | ●○ | ●○ | ●○ | ● | 7 |
| Chng *et al*., 2004 | ● | ● | ○ | ●○ | ●○ | ●○ | ● | 6 |
| Anuntaseree *et al*., 2001 | ● | ○ | ○ | ●○ | ●○ | ●○ | ● | 5 |
