# Supplementary material for: The association between allergic rhinitis and sleep: A systematic review and meta-analysis of observational studies
Source: PLoS One. 2020 Feb 13;15(2):e0228533. doi: 10.1371/journal.pone.0228533 (PMC7018032; doi:10.1371/journal.pone.0228533)
Supplement: S3 Table — (DOCX) [file pone.0228533.s024.docx]

**S3 Table. Study quality of case-control studies**

|  | **Is the case definition adequate?** | **Representativeness of the cases** | **Selection of controls** | **Definition of controls** | **Comparability of cases and controls** | **Ascertainment of exposure** | **Same method of ascertainment for cases and controls** | **Non-response rate** | **Total score** |
| --- | --- | --- | --- | --- | --- | --- | --- | --- | --- |
| Tsai *et al*., 2017 | ● | ● | ● | ● | ●○ | ● | ○ | ● | 7 |
| Nguyen-Hoang *et al*., 2017 | ● | ● | ● | ● | ●○ | ● | ● | ○ | 7 |
| Di *et al*., 2016 | ● | ● | ● | ● | ●○ | ● | ● | ● | 8 |
| Trikojat *et al*., 2015 | ● | ● | ● | ● | ●○ | ● | ● | ○ | 7 |
| Poachanukoon *et al*., 2015 | ● | ● | ● | ● | ●○ | ● | ● | ○ | 7 |
| Park *et al*., 2012 | ● | ○ | ● | ● | ●○ | ○ | ● | ○ | 5 |
| Meng *et al*., 2011 | ● | ● | ● | ● | ●○ | ● | ● | ○ | 7 |
| Rimmer *et al*., 2009 | ● | ○ | ● | ● | ●○ | ● | ● | ● | 7 |
| Sogut *et al*., 2005 | ● | ● | ● | ● | ●○ | ● | ● | ○ | 7 |
| Loekmanwidjaja *et al*., 2018 | ● | ○ | ● | ● | ●○ | ● | ● | ○ | 6 |
