# Supplementary material for: The association between allergic rhinitis and sleep: A systematic review and meta-analysis of observational studies
Source: PLoS One. 2020 Feb 13;15(2):e0228533. doi: 10.1371/journal.pone.0228533 (PMC7018032; doi:10.1371/journal.pone.0228533)
Supplement: S4 Table — (DOCX) [file pone.0228533.s025.docx]

**S4 Table. Study quality of cohort studies**

|  | **Representativeness of the exposed cohort** | **Selection of the non-exposed cohort** | **Ascertainment of exposure** | **Demonstration that outcome of interest was not present at start of study** | **Comparability of cohorts** | **Assessment of outcome** | **Was follow-up long enough for outcomes to occur** | **Adequacy of follow-up of cohorts** | **Total score** |
| --- | --- | --- | --- | --- | --- | --- | --- | --- | --- |
| Ng *et al*., 2014 | ● | ● | ● | ● | ●○ | ● | ● | ● | 8 |
| Zhang *et al*., 2012 | ● | ● | ● | ● | ●○ | ● | ● | ● | 8 |
| Dixon *et al*., 2006 | ○ | ● | ● | ● | ●○ | ● | ● | ○ | 6 |
| Lai *et al*., 2018 | ● | ● | ● | ● | ●○ | ● | ● | ● | 8 |
