# Supplementary material for: The association between allergic rhinitis and sleep: A systematic review and meta-analysis of observational studies
Source: PLoS One. 2020 Feb 13;15(2):e0228533. doi: 10.1371/journal.pone.0228533 (PMC7018032; doi:10.1371/journal.pone.0228533)
Supplement: S5 Table — AHI: apnea-hypopnea index; CI: confidence interval; ESS: Epworth Sleepiness Scale; MD: mean difference; REM: rapid eye movement; PSQI: Pittsburgh Sleep Quality Index; PSG: polysomnography. (DOCX) [file pone.0228533.s026.docx]

**S5 Table. Summary of findings for continuous outcomes.**

| Outcomes | Relative effect (95% CI) | № of participants  (studies) | Certainty of the evidence (GRADE) | Comments |
| --- | --- | --- | --- | --- |
|  |  |  |  |  |
| sleep duration | **MD 0.79** (-14.90 to 16.48) | 240,006,641  (9 observational studies) | ⨁⨁◯◯ LOW ^a,b,c,d^ | a. Did not consider confounding factors  b. Different study designs were incorporated  c. Self-reported sleep outcome  d. Different age group were incorporated |
|  |  |  |  |  |
| Sum PSQI score | **MD 0.68** (0.20 to 1.15) | 1194 (4 observational studies) | ⨁◯◯◯ VERY LOW ^a,b,c,d^ | a. Did not consider confounding factors  b. Different study designs were incorporated  c. Self-reported sleep outcome  d. Different age group were incorporated |
|  |  |  |  |  |
| PSQI-Daytime dysfunction | **MD 0.01** (-0.77 to 0.80) | 370  (2 observational studies) | ⨁◯◯◯ VERY LOW ^a,b,c,d^ | a. Did not consider confounding factors  b. Different study designs were incorporated  c. Self-reported sleep outcome  d. Different age group were incorporated |
| PSQI-Habitual sleep efficiency | **MD 0.03** (-0.26 to 0.32) | 370  (2 observational studies) | ⨁◯◯◯ VERY LOW ^a,b,c,d^ | a. Did not consider confounding factors  b. Different study designs were incorporated  c. Self-reported sleep outcome  d. Different age group were incorporated |
|  |  |  |  |  |
| PSQI-Sleep disturbance | **MD 0.20** (0.13 to 0.27) | 370  (2 observational studies) | ⨁◯◯◯ VERY LOW ^a,b,c,d^ | a. Did not consider confounding factors  b. Different study designs were incorporated  c. Self-reported sleep outcome  d. Different age group were incorporated |
|  |  |  |  |  |
| PSQI-Sleep latency | **MD 0.29** (0.13 to 0.45) | 370 (2 observational studies) | ⨁◯◯◯ VERY LOW ^a,b,c,d^ | a. Did not consider confounding factors  b. Different study designs were incorporated  c. Self-reported sleep outcome  d. Different age group were incorporated |
|  |  |  |  |  |
| PSQI-Sleep quality | **MD 0.19** (-0.40 to 0.78) | 370 (2 observational studies) | ⨁◯◯◯ VERY LOW ^a,b,c,d^ | a. Did not consider confounding factors  b. Different study designs were incorporated  c. Self-reported sleep outcome  d. Different age group were incorporated |
|  |  |  |  |  |
| PSQI-Use of sleep medications | **MD -0.07** (-0.16 to 0.01) | 370 (2 observational studies) | ⨁◯◯◯ VERY LOW ^a,b,c,d^ | a. Did not consider confounding factors  b. Different study designs were incorporated  c. Self-reported sleep outcome  d. Different age group were incorporated |
|  |  |  |  |  |
| ESS score | **MD 1.53** (-0.23 to 3.30) | 1,449 (3 observational studies) | ⨁◯◯◯ VERY LOW ^a,b,c,d^ | a. Did not consider confounding factors  b. Different study designs were incorporated  c. Self-reported sleep outcome  d. Different age group were incorporated |
|  |  |  |  |  |
| PSG-AHI | **MD 0.91** (-2.79 to 4.61) | 883 (5 observational studies) | ⨁◯◯◯ VERY LOW ^a,b,d^ | a. Did not consider confounding factors  b. Different study designs were incorporated  d. Different age group were incorporated |
|  |  |  |  |  |
| Oxyhemoglobin saturation (%) | **MD 0.99** (-0.99 to 2.97) | 247  (2 observational studies) | ⨁◯◯◯ VERY LOW ^a,d^ | a. Did not consider confounding factors  d. Different age group were incorporated |
|  |  |  |  |  |
| PSG-Sleep efficiency | **MD -3.95** (-7.00 to -0.45) | 771 (4 observational studies) | ⨁◯◯◯ VERY LOW ^a,b,d^ | a. Did not consider confounding factors  b. Different study designs were incorporated  d. Different age group were incorporated |
|  |  |  |  |  |
| Sleep stage 1 (%) | **MD -4.88** (-16.46 to 6.70) | 471 (2 observational studies) | ⨁◯◯◯ VERY LOW ^a,b^ | a. Did not consider confounding factors  b. Different study designs were incorporated |
|  |  |  |  |  |
| Sleep stage 2 (%) | **MD -0.63** (-3.24 to 1.99) | 472 (2 observational studies) | ⨁◯◯◯ VERY LOW ^a,b^ | a. Did not consider confounding factors  b. Different study designs were incorporated |
|  |  |  |  |  |
| Sleep stage 3 (%) | **MD 0.38** (-2.98 to 3.73) | 472 (2 observational studies) | ⨁◯◯◯ VERY LOW ^a,b,d^ | a. Did not consider confounding factors  b. Different study designs were incorporated  d. Different age group were incorporated |
|  |  |  |  |  |
| Sleep stage REM (%) | **MD -1.39** (-3.04 to 0.26) | 755 (4 observational studies) | ⨁◯◯◯ VERY LOW ^a,b,d^ | a. Did not consider confounding factors  b. Different study designs were incorporated  d. Different age group were incorporated |
|  |  |  |  |  |
