# Supplementary material for: The association between allergic rhinitis and sleep: A systematic review and meta-analysis of observational studies
Source: PLoS One. 2020 Feb 13;15(2):e0228533. doi: 10.1371/journal.pone.0228533 (PMC7018032; doi:10.1371/journal.pone.0228533)
Supplement: S6 Table — CI: confidence interval; MD: mean difference; OR: odds ratio; OSA: obstructive sleep apnea; SD: standard deviation; SDB: sleep-disordered breathing. (DOCX) [file pone.0228533.s027.docx]

**S6 Table. Summary of findings for dichotomous outcomes.**

| Outcomes | Relative effect (95% CI) | № of participants  (studies) | Certainty of the evidence (GRADE) | Comments |
| --- | --- | --- | --- | --- |
|  |  |  |  |  |
| Insomnia | **OR 1.84** (1.07 to 3.16) | 5,556  (1 observational study) | ⨁⨁◯◯ LOW ^a,b,d^ | a. Single study  b. AR was self-reported  d. Sleep outcome was self-reported |
| Nocturnal sweating | **OR 3.82** (0.35 to 41.24) | 6,556  (2 observational studies) | ⨁◯◯◯ VERY LOW ^b,c,d^ | b. AR was self-reported  c. Different study design  d. Sleep outcome was self-reported |
| Nocturnal enuresis | **OR 1.75** (1.44 to 2.13) | 664,220  (3 observational studies) | ⨁⨁◯◯ LOW ^b,c,d,e^ | b. AR was self-reported  c. Different study design  d. Sleep outcome was self-reported  e. Different age group |
|  |  |  |  |  |
| Sleep bruxism | **OR 1.76** (0.91 to 3.42) | 175 (1 observational study) | ⨁⨁◯◯ LOW ^a,d,f^ | a. Single study  d. Sleep outcome was self-reported  f. small sample size |
|  |  |  |  |  |
| Restless sleep | **OR 2.20** (1.26 to 3.82) | 8,022  (3 observational studies) | ⨁⨁◯◯ LOW ^b,c,d,e^ | b. AR was self-reported  c. Different study design  d. Sleep outcome was self-reported  e. Different age group |
|  |  |  |  |  |
| SDB | **OR 3.55** (1.03 to 12.31) | 523 (2 observational studies) | ⨁◯◯◯ VERY LOW ^b,c,d,e,f^ | b. AR was self-reported  c. Different study design  d. Sleep outcome was self-reported  e. Different age group  f. small sample size |
|  |  |  |  |  |
| OSA | **OR 2.09** (1.41 to 3.10) | 11,943  (7 observational studies) | ⨁⨁◯◯ LOW ^b,c,d,e^ | b. AR was self-reported  c. Different study design  d. Sleep outcome was self-reported  e. Different age group |
|  |  |  |  |  |
| Snoring | **OR 2.34** (1.68 to 3.28) | 32,981 (9 observational studies) | ⨁⨁◯◯ LOW ^b,c,d,e^ | b. AR was self-reported  c. Different study design  d. Sleep outcome was self-reported  e. Different age group |
|  |  |  |  |  |
| Difficulty to wake up | **OR 2.58** (1.36 to 4.89) | 175 (1 observational study) | ⨁◯◯◯ VERY LOW ^a,b,d,f^ | a. Single study  b. AR was self-reported  d. Sleep outcome was self-reported  f. small sample size |
|  |  |  |  |  |
| Daytime sleepiness | **OR 1.85** (1.14 to 3.00) | 4,074  (3 observational studies) | ⨁◯◯◯ VERY LOW ^b,c,d,e^ | b. AR was self-reported  c. Different study design  d. Sleep outcome was self-reported  e. Different age group |
|  |  |  |  |  |
| Morning headache | **OR 6.16** (2.48 to 15.27) | 175 (1 observational study) | ⨁◯◯◯ VERY LOW ^a,d,f^ | a. Single study  d. Sleep outcome was self-reported  f. small sample size |
|  |  |  |  |  |
| Use of sleep medicine | **OR 1.69** (1.20 to 2.38) | 5,556  (1 observational study) | ⨁◯◯◯ VERY LOW ^a,b,d^ | a. Single study  b. AR was self-reported  d. Sleep outcome was self-reported |
